# Directing Intrinsic Chirality in Gold Nanoclusters: Preferential Formation of Stable Enantiopure Clusters in High Yield and Experimentally Unveiling the “Super” Chirality of Au_144_

**DOI:** 10.1021/acsnano.3c06568

**Published:** 2023-10-08

**Authors:** Vera Truttmann, Adea Loxha, Rareş Banu, Ernst Pittenauer, Sami Malola, María Francisca Matus, Yuchen Wang, Elizabeth A. Ploetz, Günther Rupprechter, Thomas Bürgi, Hannu Häkkinen, Christine Aikens, Noelia Barrabés

**Affiliations:** † Institute of Materials Chemistry, TU Wien, Getreidemarkt 9/E165, 1060 Vienna, Austria; ‡ Institute of Chemical Technologies and Analytics, TU Wien, Getreidemarkt 9/E164, 1060 Vienna, Austria; § Departments of Physics and Chemistry, Nanoscience Center, University of Jyväskylä, FI-40014 Jyväskylä, Finland; ⊥ Department of Chemistry, Kansas State University, Manhattan, Kansas 66506, United States of America; ¶ Department of Physical Chemistry, University of Geneva, 30 Quai Ernest-Ansermet, 1211 Geneva 4, Switzerland

**Keywords:** chirality, metal nanoclusters, ligand, gold, yield, intrinsically chiral, density functional theory

## Abstract

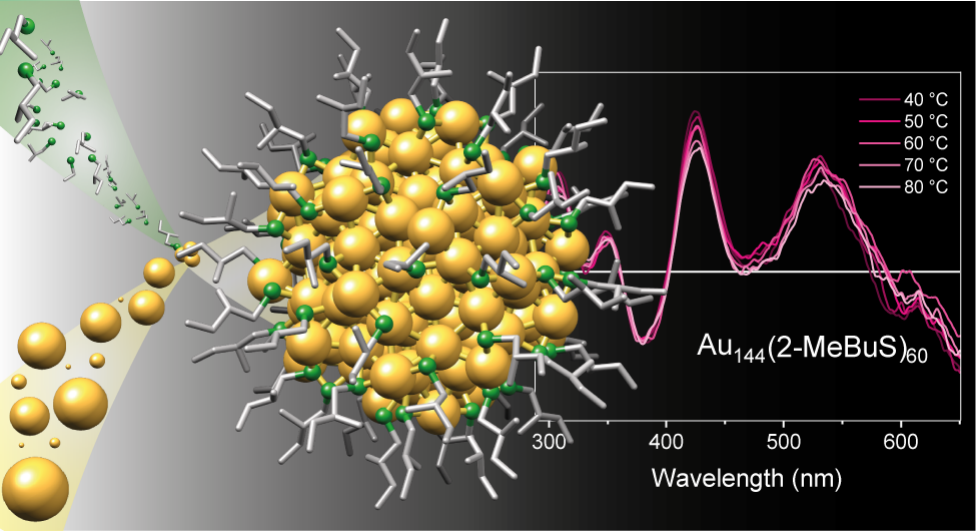

Chiral gold nanoclusters offer significant potential for exploring chirality at a fundamental level and for exploiting their applications in sensing and catalysis. However, their widespread use is impeded by low yields in synthesis, tedious separation procedures of their enantiomeric forms, and limited thermal stability. In this study, we investigated the direct synthesis of enantiopure chiral nanoclusters using the chiral ligand 2-MeBuSH in the fabrication of Au_25_, Au_38_, and Au_144_ nanoclusters. Notably, this approach leads to the unexpected formation of intrinsically chiral clusters with high yields for chiral Au_38_ and Au_144_ nanoclusters. Experimental evaluation of chiral activity by circular dichroism (CD) spectroscopy corroborates previous theoretical calculations, highlighting the stronger CD signal exhibited by Au_144_ compared to Au_38_ or Au_25_. Furthermore, the formation of a single enantiomeric form is experimentally confirmed by comparing it with intrinsically chiral Au_38_(2-PET)_24_ (2-PET: 2-phenylethanethiol) and is supported theoretically for both Au_38_ and Au_144_. Moreover, the prepared chiral clusters show stability against diastereoisomerization, up to temperatures of 80 °C. Thus, our findings not only demonstrate the selective preparation of enantiopure, intrinsically chiral, and highly stable thiolate-protected Au nanoclusters through careful ligand design but also support the predicted “super” chirality in the Au_144_ cluster, encompassing hierarchical chirality in ligands, staple configuration, and core structure.

Chirality, a property exhibited by compounds, has captivated scientific interest ever since its discovery in the 19th century.^1^ The importance of single enantiomers, particularly in pharmaceutical production, cannot be overstated due to their potentially distinct behaviors. Consequently, the development of chiral nanomaterials has become crucial in various fields such as catalysis, sensing, and medicine.^2^ Achieving chirality transfer from the molecular scale to the nanoscale holds immense promise and can greatly benefit from the utilization of well-defined chiral nanomaterials.^3−5^ Such materials would facilitate the exploration of their chiral properties while providing valuable insights into the intricate nature of molecular chirality.

Chiral ligand protected metal nanoclusters are an emerging class of atomically precise nanomaterials that distinguish themselves through their molecular to metallic properties, atomically resolved crystal structures, and ability to hold chirality at different levels.^6−9^ An important subclass, monolayer-protected gold clusters, consists of a Au core stabilized by a ligand monolayer composed of thiolates^6,7,10,11^ or other types of ligands like carbenes, alkynyls, halides, and phosphines^6,11^ or mixtures thereof. In general, imparting chirality to the nanocluster is possible by introducing chiral protecting ligands,^12,13^ which was noticed by Whetten et al. with l-glutathione (GSH) protected gold nanoclusters more than 20 years ago.^14^ In addition to this kind of embedded chirality by chiral ligands, several different Au nanoclusters can exhibit chiral properties when protected only by achiral ligands, which has become known as intrinsic chirality and was observed for the crystal structures of Au_102_(SR)_44_ and Au_38_(SR)_24_.^15,16^ The number of intrinsically chiral Au_*n*_(SR)_*m*_ nanoclusters is increasing nowadays: Au_20_(SR)_16_, Au_28_(SR)_20_, Au_38_(SR)_24_, Au_102_(SR)_44_, Au_133_(SR)_52_, and Au_144_(SR)_60_ are all proven to be chiral, despite all their thiolate ligand SRs being achiral.^6,9,15−21^

Taking a closer look at the structure of thiolate-protected gold nanoclusters, it is apparent that they are composed of a metal core protected by −(S(R)–Au)_*x*_–S(R)– units of different lengths (*x* = 1, 2, 3,...).^7^ Therefore, the intrinsic chirality in thiolate-protected gold clusters can have different origins, such as chiral arrangement of the Au–S interface or a chiral Au kernel.^6,8,12,13,21,22^ Moreover, a chiral arrangement of the organic part of the ligands can be detected in several structures, although it has, to the best of our knowledge, never been identified as the sole origin of intrinsic chirality. Combinations of more than one chiral element in a nanocluster structure have become known as hierarchical chirality.^12,13^ An extended and detailed study on the different chiral nanoclusters by Jin’s group suggested that the bonding of the ligands on the Au surface, i.e., metal–ligand interface, is responsible for most of the intrinsic chirality in thiolate protected Au nanoclusters.^8^ Unfortunately, a common problem is that the synthesis of intrinsically chiral nanoclusters produces a racemic mixture when using an achiral ligand.^12,23^

In order to obtain enantiopure forms of chiral nanoclusters, three main approaches are possible: (i) direct synthesis with chiral ligands, (ii) postsynthetic introduction of chiral ligands by ligand exchange reactions, or (iii) separation of racemic mixtures of intrinsically chiral clusters.^12^ The latter was accomplished by Bürgi and co-workers, who achieved enantioseparation of Au_38_(2-PET)_24_ (2-PET: 2-phenylethanethiol) by using chiral high-performance liquid chromatography (HPLC).^23^ Au_38_(2-PET)_24_ displayed strong signals in circular dichroism (CD) spectroscopy, which is due to the chiral arrangement of the −(S–Au)_2_–S– units on both ends of the biicosahedral Au_23_ kernel.^9,16,24^ The CD spectra of the enantiomers exhibited perfect mirror image relationships,^23^ were in good agreement with density functional theory (DFT) predictions,^25^ and enabled the assignment of the right- and left-handed enantiomers. However, in general, tailored techniques such as chromatographic separation^23^ or chiral phase transfer methods^26^ need to be developed for each chiral nanocluster composition, which can be a time-consuming and complex process.

When comparing direct synthesis and ligand exchange, it is worth noting that both approaches can be used to either induce chirality to achiral clusters or enhance their intrinsic chiral properties.^10^ The use of ligand exchange is more versatile in terms of sterics^7,10^ and solubility^10^ of the ligands. One example would be the introduction of bidentate chiral thiol ligands, 1,1′-binaphthyl-2,2′-dithiol (BINAS), to Au_25_^27−29^ and Au_38_^30^ nanoclusters. In addition, a change in the intrinsically chiral properties and thus symmetry breaking of an originally racemic Au_38_(2-PET)_24_ mixture after ligand exchange with BINAS has been reported recently.^31^ However, clusters with mixed ligand shell after ligand exchange are common,^7,10,27,32,33^ as are size and/or structure transformations of the Au nanoclusters, which makes controlling the process with atomic precision difficult.^6,10,34−36^

These complications can be avoided by applying the chiral ligand directly to the synthesis process. For thiolate-protected Au nanoclusters, adapted Brust procedures are mostly applied.^6,11,37,38^ Several factors are known to influence the outcome of a synthesis, including the solvent,^6,11^ the type of ligand,^6,7,11,37^ the kinetics of the Au_*x*_(SR)_*y*_ formation,^6,7,37,39^ or the size focusing step after reduction.^6,7,37,39^ By optimizing these factors, a number of chiral nanoclusters can be obtained almost monodispersely nowadays.^6,11^ Nevertheless, the preparation of Au nanoclusters typically suffers from relatively low yields, making the practical applicability of these materials difficult.^40,41^ Thus, several attempts have been made in the last years to develop high-yield Au nanocluster synthesis protocols.^40−43^

Direct synthesis of chiral Au nanoclusters with induced chirality has, for example, been reported for Au_25_(2-PPT)_18_ and biicosahedral [Au_25_(2-PPT)_5_(PPh_3_)_10_Cl_2_]^2+^ (2-PPT: 2-phenylpropanethiol).^44^ Both clusters exhibited CD signals up to at least 500 nm, which can be ascribed to mixing of orbitals of the chiral protecting ligands with those of the achiral Au kernel.^44,45^ If the synthesis is carried out with an intrinsically chiral cluster instead, one enantiomeric form of the cluster can be obtained, thus avoiding postsynthetic separation of enantiomers. This has been demonstrated by Jin and co-workers for Au_38_(SR)_24_ nanoclusters protected by 2-PPT, captopril, and l-glutathione.^46^ They further reported a strong influence of the chiral ligand on the CD signal, which illustrates the importance of considering all levels of chirality in Au nanocluster systems.^46^ The significant effect of different chiral ligands on the chiral properties of Au nanoclusters has also been reported for Au_11_^47,48^ and Au_25_.^49^ Although several studies have shown the critical influence of ligands and the different approaches to obtain chiral nanoclusters, in terms of practical applications, high yields in combination with strong chiroptical activity have still not been achieved.^8^

Another critical aspect that hinders the widespread application of chiral clusters is their susceptibility to racemization, which compromises their stability. The racemization process is influenced by factors such as the size, structure, and metal composition of the metal core, as well as the intricate details of the metal–thiolate interface.^50−52^ To address this challenge, one possible approach involves reducing the flexibility of the Au–S interface through the introduction of bidentate ligands.^53,54^ Furthermore, the success of this strategy is solely dependent on the ligands, and it necessitates the presence of intrinsically chiral clusters with high barriers to racemization. Theoretical calculations conducted by Malola and Hakkinen predicted that Au_144_(SCH_2_Ph)_60_ would exhibit the strongest intrinsically chiral properties and possess high stability against racemization. However, to the best of our knowledge, no experimental evidence was available at the time, due to the challenges associated with obtaining enantiopure Au_144_ nanoclusters.^50,51^ Early experimental approaches to obtain enantiopure Au_144_ involved the synthesis with alkynyl ligands, resulting in Au_144_(CCPhF)_60_ clusters.^55^ However, the observed circular dichroism (CD) signal of Au_144_(CCPhF)_60_ solely originated from the 30 FPhCC-Au-CCPhF units on the utmost surface of the achiral Au_144_ core. In contrast, the calculation of the Au_144_(SCH_2_Ph)_60_ cluster revealed that the chirality extended deeper into the cluster core, resulting in a CD response similar to those of the extensively investigated chiral clusters.

To overcome the challenges discussed above, we present this study, wherein we successfully synthesized highly pure chiral Au_25_, Au_38_, and Au_144_ nanoclusters using the chiral thiol ligand (*S*)-2-methylbutanethiol (2-MeBuSH). Notably, the yield of intrinsically chiral clusters, specifically Au_38_ and Au_144_, was significantly enhanced compared to syntheses employing butanethiol (HS-Bu) or 2-phenylethanethiol (2-PET) as ligands. The confirmation of cluster formation with an excess of a single enantiomer was achieved by comparing the circular dichroism (CD) spectra of Au_38_(2-MeBuS)_24_ and Au_144_(2-MeBuS)_60_ with previously reported data from other authors. Furthermore, our findings show that the chiral properties of these clusters are predominantly preserved even upon heating to 80 °C with no significant decrease in the CD signal. This provides compelling evidence that the 2-MeBuSH ligand enables the preferential production of highly stable and nearly enantiopure chiral clusters in substantial yields. Notably, our experimental observations align well with the calculated results and shed light on the dynamic behavior of the cluster structure.

## Results and Discussion

### Chiral Nanocluster Synthesis with 2-MeBuSH

**Figure 1:**
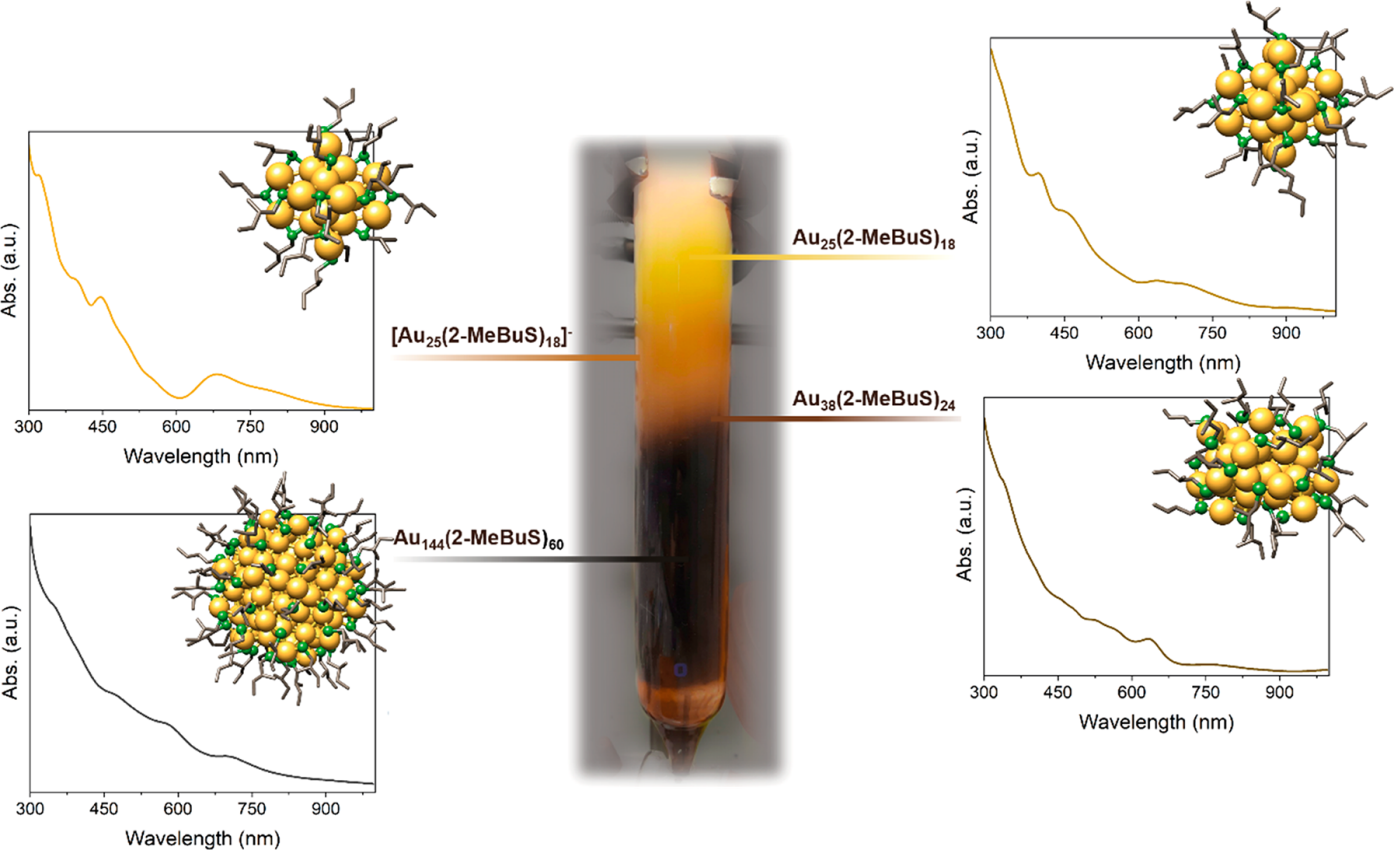
Exemplary picture of size exclusion separation after a Au_144_(2-MeBuS)_60_ synthesis and corresponding UV–vis spectra of the isolated products. The insets show exemplary structures of the (*S*)-2-MeBuS-protected Au nanoclusters. Note that the enantiomers chosen in the representation of Au_38_(2-MeBuS)_24_ and Au_144_(2-MeBuS)_60_ are not necessarily the enantiomers formed during synthesis.

Two clusters known to be intrinsically chiral, Au_38_(2-MeBuS)_24_ and Au_144_(2-MeBuS)_60_, were prepared following published direct synthesis protocols, using the chiral (*S*)-2-MeBuSH ligand (see the Supporting Information).^56,57^ In addition, [Au_25_(2-MeBuS)_18_]TOA, which does not exhibit intrinsically chiral features, was prepared for comparison.^58^ The clusters were characterized by ultraviolet–visible (UV–vis) spectroscopy (see Figures 1 and S4) and matrix-assisted laser desorption-ionization mass spectrometry (MALDI-MS) (Figures S5–S7).

Despite following reported synthesis protocols using common thiolate ligands for each cluster, significant differences in product distribution were observed when using the chiral (*S*)-2-MeBuSH ligand. Figure 1 illustrates the size exclusion chromatography analysis of the crude product obtained after Au_144_ synthesis with 2-MeBuSH as the ligand, revealing the presence of four distinct cluster sizes. The primary fraction corresponds to Au_144_, accompanied by smaller amounts of Au_38_, as well as both neutral and anionic Au_25_ clusters eluting subsequently. The presence of Au_25_ as a common byproduct in the synthesis of Au_144_ has been previously reported by Qian et al.^56^ and was also observed in our study when employing 2-PET or HS-Bu as ligands. However, employing the chiral ligand 2-MeBuSH resulted in the additional formation of intrinsically chiral Au_38_ clusters in significant quantities. Interestingly, unexpected product distributions were also observed in the synthesis of Au_25_ and Au_38_ using 2-MeBuSH. A considerable amount of Au_144_ was obtained as a side product, whereas no Au_144_ formation was observed when HS-Bu was used as the ligand. Furthermore, although the anionic form remained the predominant product in the synthesis of Au_25_, a higher proportion of the neutral species was obtained compared to conventional synthesis conditions. The purity of the synthesized clusters was evaluated by UV–vis spectroscopy (Figure S4) as well as MALDI-MS analysis (Figures S5–S7).

Moreover, we also observed a significantly enhanced yield of pure chiral clusters during the synthesis of both Au_38_ and Au_144_ when employing 2-MeBuSH, surpassing the reported yields achieved with similar achiral ligands.^40,41,56^ Specifically, the use of 2-MeBuSH led to a noteworthy yield of 78% for Au_38_, in contrast to 26% obtained with HS-Bu (which differs from 2-MeBuSH by the absence of a methyl group on the β-carbon atom). Similarly, for Au_144_, the yield reached 73% with 2-MeBuSH compared with 34% with HS-Bu. However, this trend was not observed for the achiral Au_25_ cluster as similar yields were obtained with both ligands (25% for 2-MeBuSH and 23% for HS-Bu). Thus, the preferential formation of intrinsically chiral clusters, facilitated by the chiral environment provided by the chiral ligand, was evident, leading to high yields of enantiopure clusters. This observation can be attributed to differences in the arrangement of the two enantiomers of the chiral ligands on the surface of the Au core.^59^

These findings highlight the preferential formation of intrinsically chiral Au nanoclusters using 2-MeBuSH as the chiral ligand in the synthesis. The observed increased yield and shift toward intrinsically chiral clusters suggest a hierarchical chiral inheritance during the synthesis, whereby one chiral center influences another, an effect commonly observed in chemistry and biology but not yet extensively documented with Au nanoclusters.

### Chirality of 2-MeBuSH Protected Au Nanoclusters

**Figure 2:**
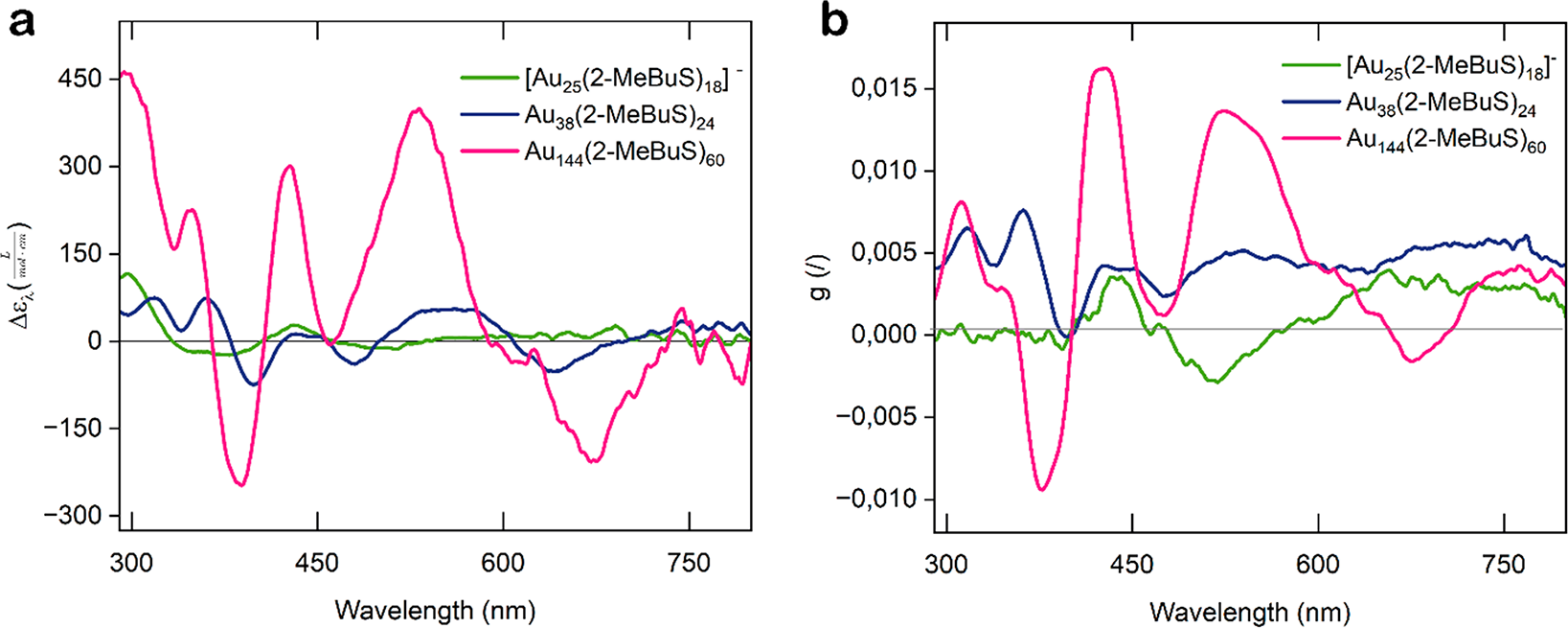
(a) Circular dichroism (CD) spectra of the [Au_25_(2-MeBuS)_18_]^−^, Au_38_(S-MeBuS)_24_, and Au_144_(S-MeBuS)_60_ nanoclusters at RT. To obtain a concentration-independent signal, the molar extinction coefficients (Δε_λ_) of the three clusters were calculated by , with θ as the CD signal, *c* as the molar concentration, and *d* as the path length. (b) Anisotropy factor *g*, calculated as  of the three nanoclusters.

The chiral properties of the 2-MeBuSH protected Au nanoclusters were studied by CD spectroscopy (Figure 2a). The spectra are very different from those of the chiral 2-MeBuSH ligand (Figure S9), confirming that the structural chirality is not only located in the ligand. Both Au_38_(2-MeBuS)_24_ and Au_144_(2-MeBuS)_60_ showed low-energy signals above 500 nm, consistent with the CD signal being majorly influenced by the structural arrangement of the Au atoms.^45^ This was also observed for [Au_25_(2-MeBuS)_18_]^−^, however, with significantly lower intensity compared to the intrinsically chiral clusters. Nevertheless, a contribution from the core orbitals also seems likely for this intrinsically achiral cluster, since the signals appear significantly red-shifted with respect to those of the free ligand (Figures 2a and S9).^44^

The Au_144_(2-MeBuS)_60_ cluster exhibited the strongest signal, followed by Au_38_(2-MeBuS)_24_, and finally [Au_25_(2-MeBuS)_18_]^−^. Considering the intrinsically chiral nature of Au_144_ and Au_38_ clusters, this can be attributed to a cooperative effect between the ligand and the arrangement of core/units.^16,23,25^

In the case of Au_38_, its chiral properties primarily arise from the Au–S interface, where the −(S(R)–Au)_2_–S(R)– motifs self-assemble into a chiral pattern. Similarly, Au_144_ features five rings of monomeric units, with the orientation of these units imparting chirality to the structure.^20,60,61^ Previous DFT calculations^50,51,62^ have predicted the strong chiral properties of Au_144_, even suggesting that the core itself contributes to a noticeable CD signal.^51^ The stronger signal of Au_144_ can be attributed to the chiral arrangement of the 30 units on the core surface, which amplifies the inherent chirality originating from the core itself.^51^ Furthermore, compared to Au_38_, Au_144_ has been predicted to exhibit significantly greater stability against rearrangement procedures that could lead to inversion of chirality, thereby preserving the enantiopurity of the cluster samples.^50^

### Handedness and Enantiopurity

#### Au_38_(2-MeBuS)_24_

**Figure 3:**
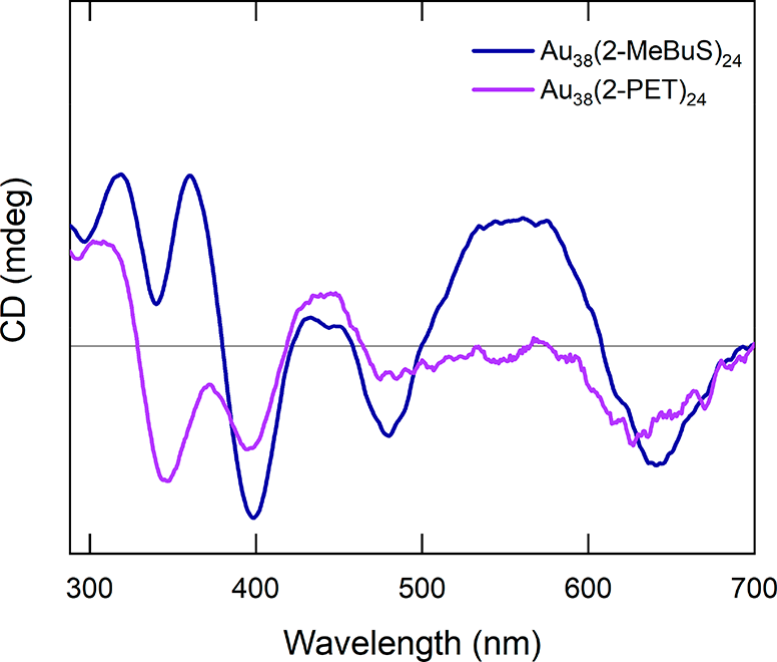
Comparison of the CD spectra of Au_38_(2-MeBuS)_24_ and the enantiomer of Au_38_(2-PET)_24_ eluting first during a HPLC separation following the procedure by Bürgi and co-workers.^23^

In order to experimentally determine the enantiomer handedness and assess its purity, a comparison approach was employed, based on the successful separation of the two enantiomers of Au_38_(2-PET)_24_ achieved by Bürgi and co-workers.^23^ This allowed the investigation of the chiral properties of Au_38_(2-MeBuS)_24_. Following the separation protocol reported by Bürgi and co-workers, Au_38_(2-PET)_24_ was synthesized and subjected to chiral HPLC separation (Figure S8). By comparing the CD spectrum of Au_38_(2-MeBuS)_24_ with those of the two enantiomers of Au_38_(2-PET)_24_ (Figure 3), it can be observed that the enantiomer of Au_38_(2-PET)_24_ eluting first during the HPLC separation exhibits strong similarities to the CD spectrum of Au_38_(2-MeBuS)_24_. This suggests that this particular enantiomer was preferentially obtained during the synthesis with (*S*)-2-MeBuSH.

To further confirm this, a chiral HPLC separation of Au_38_(2-MeBuS)_24_ was performed using the published method for the separation of Au_38_(2-PET)_24_.^23^ The corresponding chromatograms of both separations are presented in Figure S10. In the case of Au_38_(2-MeBuS)_24_, only one major peak is observed at a retention time of 2 min, whereas the racemic Au_38_(2-PET)_24_ exhibits two peaks at 11.3 and 23.2 min. The differences in retention time between Au_38_(2-MeBuS)_24_ and the first enantiomer of Au_38_(2-PET)_24_ can be attributed to the influence of the respective ligand. These results further indicate that enantiopure Au_38_(2-MeBuS)_24_ was obtained through the synthesis with a chiral ligand.

In the case of Au_38_(2-MeBuS)_24_, the experimental CD spectrum was first compared with previous calculations on Au_38_(SCH_3_)_24_,^25^ which indicates that the obtained isomer is the one showing an anticlockwise rotatory arrangement of the −S(R)–[Au–S(R)]_*x*_– units. Then, the CD spectrum of Au_38_(2-MeBuS)_24_ was also simulated by means of DFT. Model structures of Au_38_(2-MeBuS)_24_ were made, based on both the Au_38_(2-PET)_24_ crystal structure^16^ (isomer 1), as well as on the lowest energy structure of Au_38_(SCH_3_)_24_ found by Lopez-Acevedo et al. (isomer 2), namely, structure 1 in their work.^25^ To avoid confusion, this structure 1 will be referenced as the *JACS2010* structure in the following discussion. For both structures investigated (isomer 1 and isomer 2), both an anticlockwise (denoted with an *a*) and a clockwise (denoted with a *b*) conformer were created and optimized at the BP86/DZP level of theory,^63−65^ taking into account scalar relativistic effects^66,67^ by employing the AMS software^68^ (see the Supporting Information for further details). Further refinements of each substructure produced the lowest energy isomers, namely, 1a, 1b, 2a, and 2b, the energies of which are compared in Table S1.

It appears that the isomers obtained starting from the *JACS2010* structure proved to be lower in energy than their crystal structure-based counterparts. This might be explained by the slightly different arrangements of the ligands for the two structures, as a comparison of their structures in Figure S11 shows.^16,25^ However, as can be seen from Figure S12, the impact on the optical properties was negligible, which could be expected considering that the excited states with λ > 500 nm are not usually sensitive to the ligand conformation, because these excitations primarily originate in the metal core.^12^ Thus, further calculations focused only on isomers 2a and 2b. For these two, classical molecular dynamics (MD) simulations were carried out using the GROMACS package^69−71^ and including dichloromethane solvent molecules with GAFF^72^ (see details in the Supporting Information) for an extended isomer search.

To simulate the UV–vis and CD spectra of the lowest energy isomers, linear response TD-DFT+TB calculations^73^ in the gas phase were carried out and the spectra obtained after a Gaussian fit.^48,74^ It should be noted that TD-DFT+TB was significantly faster than standard time-dependent density functional theory (TD-DFT).^75^ Nevertheless, no significant differences were observed below 900 nm in the optical absorption and CD spectra calculated for the isomer with both methods (see Figure S13), which is why TD-DFT+TB was then applied exclusively. For excitations at wavelengths longer than 900 nm, the TD-DFT+TB excited state energies closely approximate TD-DFT, although there are some differences in rotatory strengths.

**Figure 4:**
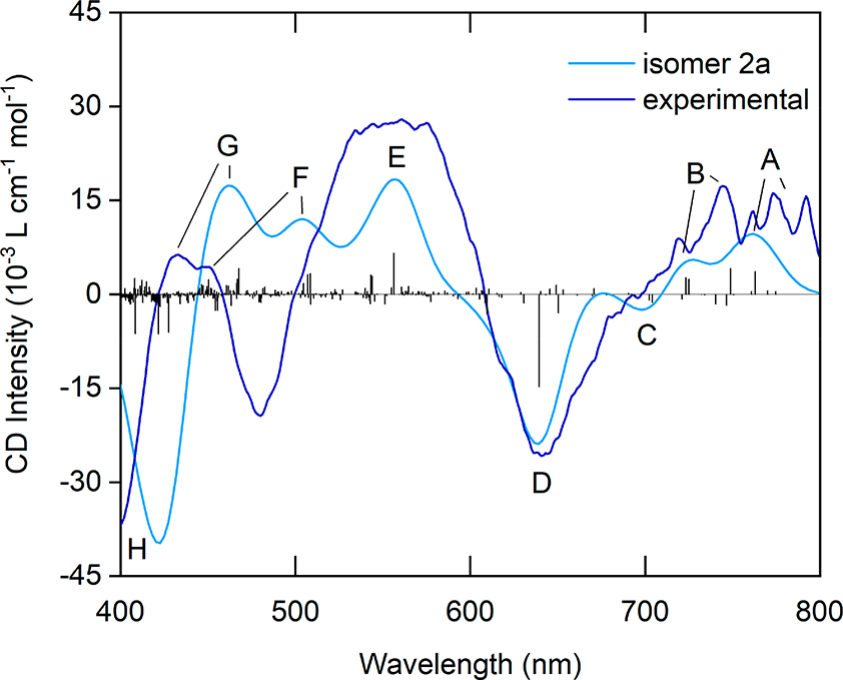
Comparison of the theoretical CD spectrum of isomer 2a in the gas phase including rotatory strength with the experimental spectrum of Au_38_(2-MeBuS)_24_. Eight distinguishable bands have been labeled and assigned.

To confirm that the anticlockwise enantiomer was indeed preferentially formed during synthesis with (*S*)-2-MeBuSH, the experimental CD spectrum of Au_38_(2-MeBuS)_24_ was compared to the calculated one of isomer 2a (Figure 4). The observed experimental and theoretical maxima are listed in Table S2. Indeed, the experimental CD spectrum mainly follows the same trend and shows similar bands with only slight deviations in the excitation energies, especially in the higher energy end of the spectra. This agrees with the experimental comparison to anticlockwise Au_38_(2-PET)_24_ (Figure 3) and confirms that this enantiomer has been formed preferentially during synthesis. Good agreement was also obtained upon comparison of the experimental and calculated UV–vis spectra of Au_38_(2-MeBuS)_24_ (Figure S14).

Finally, the influence of the intrinsic chirality of the cluster versus the influence of the chiral ligand can be evaluated when comparing the CD spectra of the lowest energy clockwise and anticlockwise isomers (2a and 2b, respectively; see their structures in Figure S15 and computed spectra in Figure S16). Since the signal in the CD spectra of Au_38_(2-MeBuS)_24_ is expected to be mainly due to the chiral Au–S interface,^25^ their CD spectra compare to mirror-images (see Figure S16b), even though these two clusters are in reality diastereomers (as opposed to a pair of enantiomers). This is further evidenced by comparing the spectra of isomer 2b to those of the *JACS2010* structure^25^ (both are clockwise isomers but have a different ligand): For both the UV–vis and the CD spectra, the shape and positions of the bands are found to be very similar within the energy range that can be compared. This indicates that the 2-MeBuSH ligand does not induce significant changes to the cluster kernel and staple geometry, which was expected owing to its small size.

#### Au_144_(2-MeBuS)_60_

However, in the case of Au_144_(2-MeBuS)_60_, the same approach was not possible. Predicted to be a cluster with very strong optical activity, Au_144_ chirality was only experimentally proved by resolved crystal structure until now.^20^ The reason for this stems from the challenging separation procedure of the two Au_144_ enantiomers from a racemic mixture. Thus, the spectrum of Au_144_(2-MeBuS)_60_ shown in Figure 2 presents important advances in the experimental elucidation of the CD spectrum of this cluster.

The strong CD response shows that the chiral ligand dictates the handedness of the chiral Au–ligand interface. The chiral thiol ligand in the synthesis clearly resulted in symmetry breaking, which resulted in at least a large enantiomeric excess of one enantiomer, thus allowing the observation of an experimental CD spectrum of the Au_144_ cluster. Whether the other diastereomer (with opposite handedness of the Au–ligand interface) is also present in small quantities is difficult to judge.

Naturally, the question of which enantiomer of the cluster is represented by the measured CD spectrum arises. Although the comparison of the measured CD data of Au_144_(2-MeBuS)_60_ to our previous theoretical predictions on Au_144_ (SCH_2_Ph)_60_^51^ indicates the presence of the right-handed enantiomer, we set out to recalculate the CD spectrum using the 2-MeBuSH ligand. To this end, we used the GPAW software^76^ (see the Supporting Information for more details). First, a model structure for Au_144_(2-MeBuS)_60_ was built by replacing the SCH_2_Ph thiolates of the Au_144_(SCH_2_Ph)_60_ crystal structure^20^ with (*S*)-2-MeBuS ligands. The model structure was optimized as a neutral system using the Perdew–Burke–Ernzerhof (PBE) exchange-correlation functional.^77^ The optimization led to a spatially extended overall size due to Au–Au bond overestimation by 2–3%, a known artifact of the PBE functional. However, the symmetrical, intrinsically chiral conformation of the cluster remained stable under optimization, as shown in Figure S17.

The optical and chiral properties were calculated using linear response time-dependent density functional theory (lr-TD-DFT)^78^ for the optimized model structure. The results of the optical absorption are shown in Figure S18, including the labeling from 1 to 5 for the observed features. The calculated optical absorption spectrum matches the experimental one well, as both spectra have a low energy tail above 700 nm. Feature 1 is located at the tail just above 700 nm experimentally and below 800 nm computationally. Features 2 and 3 are found at lower wavelengths: between 450 and 650 nm experimentally and between 500 and 700 nm computationally. Moreover, at even lower wavelengths (below 450 nm), there are two more features in both spectra, which are labeled 4 and 5. Generally, the features seen in the measured spectrum are weaker than those seen in the calculated spectrum, but the agreement is reasonable overall.

**Figure 5:**
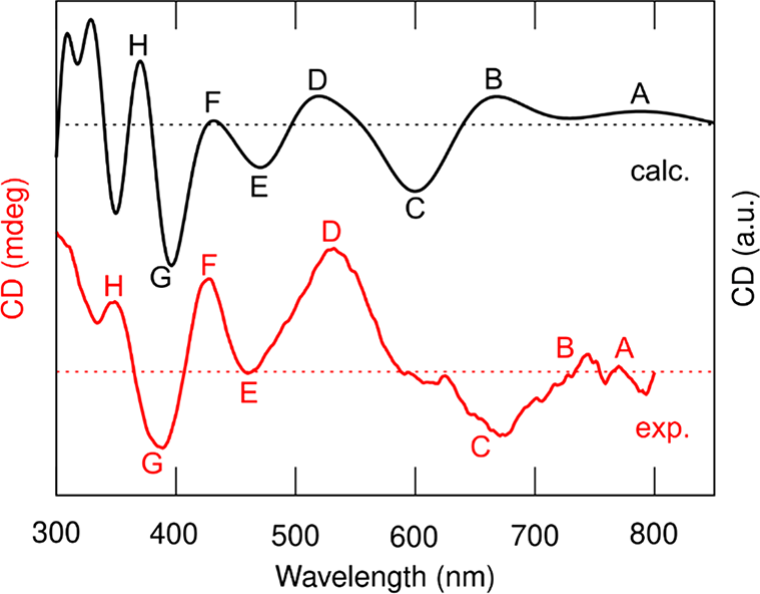
Measured CD spectrum of the Au_144_(2-MeBuS)_60_ cluster (red curve) compared to the calculated CD spectrum of the symmetrical model structure (black curve). Eight distinguishable bands have been labeled and assigned from both spectra. The calculated spectrum is scaled based on the intensity of peak C in the experimental spectrum.

The most interesting comparison is made between the calculated and measured CD spectra of Au_144_(2-MeBuS)_60_. As shown in Figure 5, the calculated spectrum has eight identifiable bands (labeled from A to H), and the same number of bands can be identified in the measured spectrum. The exact positions of the bands are listed in Table S3, showing only minor discrepancies in the positions when comparing calculated and experimental data. This strongly indicates that the synthesized clusters are indeed predominantly of the same handedness as our model (specifically, the right-handed enantiomer shown in Figure S17).

Next, we analyzed the peaks in the computed CD spectrum by using the rotatory strength transition contribution map (RTCM) method. Figure S19 reveals that the lower energy bands are due to transitions between the superatom states localized in the metal core and the metal–ligand interface states. As shown previously,^51^ the metal core and the metal–ligand interface are the main contributors to the chirality of the intrinsically chiral systems. At higher energies (wavelengths < 500 nm), the role of the ligand states as well as Au d-band states start to increase, as shown in Figure S20.

#### Thermal Stability

An important aspect when dealing with intrinsically chiral Au nanoclusters is their stability against racemization, i.e., structural reorganization processes in the core–ligand interface.^22,50,52^ This will usually affect their circular dichroism spectra, resulting in diminished features and ultimately loss of chiroptical activity.^22,52^ Thus, the temperature stability of the clusters was tested by *in situ* CD measurements at elevated temperatures. Therefore, the clusters were dissolved in toluene and heated to 80 °C *in situ* with CD spectra measured every 10 °C.

**Figure 6:**
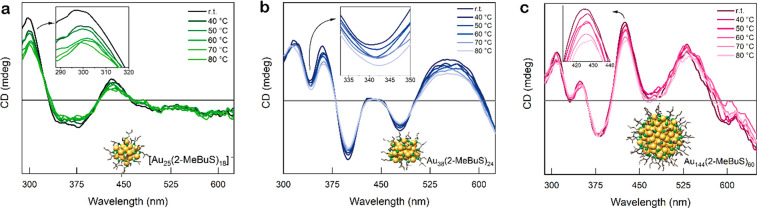
Evolution of the CD spectra with increasing temperature (RT to 80 °C): [Au_25_(2-MeBuS)_18_]^−^ (a), Au_38_(2-MeBuS)_24_ (b), and Au_144_(2-MeBuS)_60_ (c).

For Au_38_, the CD spectra measured at the different temperatures are displayed in Figure 6b. The minimum at 341 nm was chosen for quantification of the relative decrease in CD signal to be able to compare it to the data previously reported.^52^ At 40 °C, a decrease to 88% of the original intensity was observed; at 50 and 60 °C and at 70 °C, it decreased to 72% and 70%, respectively, relative to the original signal. Comparing this to the study performed by Bürgi’s group,^52^ it can be deducted that the stability against racemization of the Au_38_(2-MeBuS)_24_ cluster is higher than that of Au_38_(2-PET)_24_. By analysis of the relative intensities of the band at 345 nm, relative intensities of 97% for 40 °C, 92% for 50 C, 65% for 60 °C, and 23% for 70 °C were calculated.

It should be noted that, at low temperatures (40 and 50 °C), the 2-PET cluster shows slightly higher stability, whereas at higher temperatures (60 and 70 °C), the 2-MeBuS cluster retains a significantly stronger CD signal as compared to the 2-PET cluster. In fact, at high temperatures, the CD signal of the Au_38_(2-PET)_24_ cluster approaches zero (complete racemization) after some time. Whereas in the case of Au_38_(2-PET)_24_ the racemization involves the transformation of one enantiomer into the other, this is not the case for the cluster considered in this study (Au_38_(2-MeBuS)_24_). Here, the inversion of the configuration of the Au–S framework (also termed “racemization” above, which is in fact a diastereoisomerization) leaves the configuration of the ligand unaffected. Put in other words, the inversion of the absolute configuration of the Au–S framework leads to a diastereomer with respect to the initial structure. Normally, diastereomers have different energies (in contrast to true enantiomers), which is probably the reason for the observation of this work, i.e., that one configuration of the Au–S framework is produced in large excess (or even pure) during the synthesis when using one enantiomer of the ligand. The higher stability of one cluster diastereomer is likely also at the origin of the retained CD signal, even at higher temperatures. The increased stability of one diastereomer leads to an excess of that diastereomer at equilibrium, in contrast to the case of Au_38_(2-PET)_24_.

The different behavior depending on the temperature may reflect the different kinetics and thermodynamics of the two systems. The higher stability of the Au_38_(2-PET)_24_ cluster at low temperature may be attributed to the larger size of 2-PET compared to 2-MeBuSH. This could offer a slight steric advantage for the Au_38_(2-PET)_24_ cluster, resulting in slower kinetics of the inversion of the Au–S framework. However, the drastic decrease of CD signal of the Au_38_(2-PET)_24_ cluster at higher temperatures and the stability of the Au_38_(2-MeBuS)_24_ cluster reflect the thermodynamic stability of one diastereomer over the other in the case of Au_38_(2-MeBuS)_24_. Overall, the addition of a second chiral level through the optically active 2-MeBuSH increases the nanocluster’s stability against inversion of the Au–S framework at higher temperatures when the steric protection offered by bulkier ligands is no longer sufficient to kinetically hinder the racemization process.

The CD spectra of Au_25_(2-MeBuS)_18_ and Au_144_(2-MeBuS)_60_ at different temperatures can be found in Figure 6a,c. Regarding the stability of the Au_144_ nanocluster, the band at 425 nm was considered for the relative decrease in the CD signal. A percent decrease to 82% of the original intensity was observed at 70 °C, which is slightly lower than that of Au_38_, indicating a higher stability of the Au_144_ cluster. This is in accordance with the calculations performed by Malola et al.,^50^ who state that the energy barrier for the inversion of Au_38_ should lie lower than the one of Au_144_. Of note, the thermal energy brought to the system by heating to 70 °C should be significantly lower than their estimated activation energies.

Furthermore, the results clearly show that the changes in the relative intensity of the CD spectra of the three clusters as a function of increasing temperature are of the same order of magnitude, hence indicating a similar thermal stability. One plausible explanation is that no significant diastereoisomerization is observed below 80 °C, but the changes are due to the increased distortions and the dynamics of the ligands, as shown computationally before for the Au_144_ cluster.^50^ The average structure of the ligand layer is expected to be very close to the symmetrical arrangement, but the dynamical flipping of ligands particularly affects the UV-region signals. It appears that the 2-MeBuSH ligand not only provides high yields for enantiopure synthesis and production of intrinsically chiral clusters but also stabilizes them at elevated temperatures preserving their chiral properties.

#### Dynamics of the Ligand Shell Effect on the CD Spectra of Au_144_

Finally, the ligand shell effect on the circular dichroism spectra was also analyzed employing theory, using the Au_144_ cluster as an example. First, the effects of temperature on the dynamics of the ligand layer were studied. We performed a 300 ns classical MD simulation using the GROMACS software^71^ and our previously published force field for thiolate-protected gold nanoclusters.^79^ The DFT-optimized model of Au_144_(2-MeBuS)_60_ was used as a starting structure, which was then solvated in methanol and simulated according to the experimental conditions (see the Supporting Information for more details). Several properties related to structural integrity were analyzed by the MD trajectory. As shown in Figure S21, the fluctuation of the radius of gyration (*R*_g_) over the simulation time is minimal, oscillating between 0.775 and 0.780 nm, indicating that the overall cluster structure remains intact and stable during the MD run. Likewise, the root-mean-square deviation (RMSD) of atomic positions fluctuates in a small range, between 0.07 and 0.08 nm (Figure S21). In addition, visual inspection shows that the intrinsically chiral arrangement of the gold–thiolate units at the metal–ligand interface remains the same. Although minor distortions are observed in the individual units, the handedness of the cluster is preserved throughout the simulation. This was also expected based on previous calculations for the Au_144_ cluster that predict a high activation barrier for the inversion of the chirality by metal core reconstruction.^50^

Furthermore, the dynamics of the organic part of the ligand layer were studied via the so-called essential dynamics (ED) analysis,^80,81^ which can distinguish the most important dynamical directions of the system in the 3N-dimensional coordinate space. Projecting the dynamics onto the two main principal axes of the data, we can form conformational free energy plots using the MD trajectories, shown in Figure S22. It shows that the individual data points (each comprising one structure conformation of a MD-run) span a large area of the projection space, not forming a single basin of the free energy. The reason is found by checking the C–S···S–C dihedral angles within the protecting units. A dihedral angle of 0° means that the ligands within the same unit are pointing to the same side, while when the angle is 100° and −100°, they are pointing to different sides of the linear protecting unit. Figure S23 shows that the ligands are flipping frequently during the whole simulation; in other words, we do not find just one stable conformation of the organic layer, although the symmetries of the metal core and metal–ligand interface structures are maintained.

It is now interesting to see how much the disorder in the organic layer affects the CD signal. For that, we selected a snapshot structure around the minimum region of the conformational free energy, shown in the ED plot in Figure S22. The snapshot structure was first optimized using DFT, and then, the calculation of chiroptical properties was repeated. The absorption and CD spectra of the optimized snapshot structure are shown in Figure S24 and compared to the spectra of the optimized symmetric model structure. The shape of the optical absorption spectrum remains identifiable, but the features are smoother than those for the optimized symmetric structure. The changes observed in the CD spectrum are more distinguishable than those in the optical absorption spectra. The low energy peaks A and B merge and are slightly red-shifted. The absolute intensity of the C–F peaks gets lower, and their positions are also slightly red-shifted. The clearest differences are observed for the peaks having dominant contributions from the ligand states. The intensities of the G and H peaks drop to close to zero. Despite the more significant changes in the UV-region peaks, the handedness of the cluster remains identifiable from the CD spectrum.

## Conclusions

Using (*S*)-2-MeBuSH as a chiral thiol ligand in the synthesis of Au_144_ and Au_38_ nanoclusters led to several noteworthy findings. To begin with, it substantially increased the yield (approximately by 300%) compared to previous reports, indicating the effectiveness of employing chiral thiols in Brust-type protocols for achieving high-yield synthesis of chiral Au nanoclusters.

Of great importance is the successful synthesis of enantiomerically pure Au_144_ clusters, which allowed the experimental investigation of their chiral properties and confirmed the theoretical predictions of their strong and stable chirality. This achievement represents an important milestone in the field of chiral nanoclusters, since prior to this work only the crystal structure of Au_144_ clusters could provide experimental evidence of their chiral nature.

Moreover, the synthesis of Au_38_(S-2-MeBuS)_24_ and comparison with Au_38_(2-PET)_24_ together with theoretical calculations showed the formation of a single enantiomer instead of a pair of diastereomers, further emphasizing the chiral control achieved in this work by the use of chiral ligands.

Furthermore, the incorporation of the (*S*)-2-MeBuSH ligand introduced an additional level of chirality, enhancing the stability of the clusters against racemization at elevated temperatures. This improved thermal stability expands the potential applications of chiral nanoclusters, as they can now withstand higher temperatures without losing their enantiopurity.

Altogether, the combination of the significant increase in yield, the preferential formation of intrinsically chiral and enantiopure nanoclusters, and the enhanced thermal stability provided by chiral thiol ligands such as (*S*)-MeBuSH establishes chiral control during synthesis as a promising strategy for the high-yield and enantiopure synthesis of chiral Au nanoclusters. These findings substantially improve the feasibility of using chiral nanoclusters in various fields, including catalysis, sensing, and biomedical applications, where their chirality and enhanced stability can offer distinct advantages.

## Methods

### Synthesis

The (*S*)-enantiomer chiral thiol ligand used in the nanocluster synthesis was obtained from the corresponding (*S*)-alcohol in a two-step process adapted from Jin and co-workers.^44^ The detailed procedure is described in the Supporting Information. All nanocluster syntheses were performed following modified Brust procedures reported previously.^56−58,82^ The detailed individual procedures are described in the Supporting Information.

### Experimental Methods

UV–vis spectroscopy was performed on a UV-1600PC spectrometer using cuvettes of 1 cm path length. Different solvents (DCM, toluene, and THF) were used to dissolve the Au nanoclusters depending on the specific reaction step.

CD spectra were obtained by using a JASCO J-810 spectropolarimeter equipped with a Peltier temperature controller. Quartz glass cuvettes with a path length of 0.2 cm were used. The samples were dissolved in DCM or toluene for measurement.

MALDI-MS was conducted on a Bruker Ultraflextreme MALDI-TOF instrument equipped with an Nd:YAG laser in linear mode. Each spectrum was obtained by averaging 5000 single shots (split in packets of 500 shots). Spectra were obtained with 10% (Au_25_ and Au_38_) or 30% (Au_144_) laser power. *trans*-2-[3-(4-*tert*-Butylphenyl)-2-methyl-2-propenyliden]-malononitrile (DCTB) was used as matrix. Sample and matrix solutions were prepared in toluene.

HPLC separation experiments of the Au_38_ nanocluster stereoisomers were performed on a Shimadzu Lab Solution LC-20 A system, following a published protocol.^23^ The instrument was equipped with a chiral 5 μm Lux Cellulose-1 (250 mm × 4.6 mm, company Phenomenex) column. The nanoclusters were dissolved in toluene, and a mobile phase of 80:20 *n*-hexane:isopropanole at 2 mL/min was chosen for separation. The elution of the nanocluster fractions was observed by UV–vis detection at 380 nm.

Nuclear magnetic resonance (NMR) spectroscopy was measured on a Bruker Avance 400 MHz NMR spectrometer. Samples were dissolved in CDCl_3_, and the solvent signal was used as an internal reference. Chemical shifts relative to trimethylsilane (TMS) are reported.

### Computational Methods

Density functional theory (DFT) calculations were employed to determine geometrical structures, optical absorption spectra, and CD spectra, as described in the Supporting Information. Molecular dynamics (MD) simulations using a gold–thiolate molecular mechanics force field were employed to examine the structural dynamics of the nanoclusters. Additional information is provided in the Supporting Information.

## Supporting Information Available

Synthesis of the chiral thiol ligand (Figure S1), synthesis of the Au nanoclusters and NMR spectra of the chiral thiol (Figures S2–S3), UV–vis spectra of the Au nanoclusters (Figure S4), MALDI-MS spectra of the Au nanoclusters (Figures S5–S7), additional CD spectra (Figures S8–S9), HPLC chromatograms (Figure S10), relative energies of the calculated Au_38_(2-MeBuS)_24_ isomers (Table S1), Au_38_ structures obtained from different starting structures and their spectra (Figure S11), theoretical UV–vis and CD spectra of Au_38_(2-MeBuS)_24_ at different levels of theory (Figure S12), calculated optical absorption spectrum compared to the experimental one (Figures S13–S14), positions of the peaks observed in the measured and calculated CD spectra of Au_38_(2-MeBuS)_24_ (Table S2), structures of the different Au_38_ enantiomers and their spectra (Figures S15–S16), computational methods, model structure of Au_144_(2-MeBuS)_60_ cluster (Figure S17), comparison of calculated and experimental optical absorption spectra of Au_144_(2-MeBuS)_60_ (Figure S18), RTCM plots of calculated CD spectrum of Au_144_(2-MeBuS)_60_ (Figures S19–S20), Rg of Au_144_(2-MeBuS)_60_ and RMSD of atoms over simulation time (Figure S21), ED analysis from MD-trajectory of Au_144_(2-MeBuS)_60_ (Figure S22), analysis of ligand dynamics from MD-trajectory of Au_144_(2-MeBuS)_60_ (Figure S23), calculated CD and optical absorption spectra of MD-snapshot of Au_144_(2-MeBuS)_60_ (Figure S24), positions of the peaks observed in the experimental and calculated CD spectra of Au_144_(2-MeBuS)_60_ (Table S3), structure of [Au_25_(2-MeBuS)_18_]^−^ and its spectra (Figures S25–S26), comparison between the theoretical and experimental UV–vis and CD spectra of [Au_25_(2-MeBuS)_18_]^−^ (Figure S27), and optical absorption and CD spectra of (*S*)-2-MeBuSH (Figure S28) (PDF)
