# Supplementary material for: Directing Intrinsic Chirality in Gold Nanoclusters: Preferential Formation of Stable Enantiopure Clusters in High Yield and Experimentally Unveiling the “Super” Chirality of Au144
Source: ACS Nano. 2023 Oct 8;17(20):20376–86. doi: 10.1021/acsnano.3c06568 (PMC10604085; doi:10.1021/acsnano.3c06568)
Supplement: Supplementary file 1 — nn3c06568_si_001.pdf [file nn3c06568_si_001.pdf]

Supporting Information

Directing Intrinsic Chirality in Gold Nanoclusters: Preferential formation of stable enantiopure clusters in high yield and experimentally unveiling the “super” chirality of Au_{144}

Vera Truttmann[1,‡], Adea Loxha[1], Rares Banu[1], Ernst Pittenauer[2], Sami Malola[3], María Francisca Matus[3], Yuchen Wang[4], Elizabeth A. Ploetz[4], Günther Rupprechter[1], Thomas Bürgi[5], Hannu Häkkinen[3], Christine Aickens[4], Noelia Barrabés[1,*]

[1] Institute of Materials Chemistry, TU Wien, Getreidemarkt 9/E165 1060 Vienna, Austria

[2] Institute of Chemical Technologies and Analytics, TU Wien, Getreidemarkt 9/E164 1060 Vienna, Austria

[3] Departments of Physics and Chemistry, Nanoscience Center, University of Jyväskylä, FI-40014 Jyväskylä, Finland

[4] Department of Chemistry, Kansas State University, Manhattan, Kansas 66506, United States of America

[5] Department of Physical Chemistry, University of Geneva, 30 Quai Ernest-Ansermet, 1211 Geneva 4, Switzerland

‡ Current address: Institute for Chemical Technology and Polymer Chemistry, Karlsruhe Institute of Technology, Engesserstraße 20, 76131 Karlsruhe, Germany

1 Synthetic Procedures

1.1 Materials

The reagents, solvents and other consumables used in the preparation of both ligands and nanoclusters were obtained from commercial suppliers. Any steps involving aqueous solutions were performed using ultrapure Milli-Q H_2O (18.6 MΩ·cm at 25 °C).
Hydrogen tetrachloroaurate trihydrate, $HAuCl_4 \cdot 3\,H_2O$ (≥49.0% Au basis), sodium tetrahydroborate, $NaBH_4$ (98%), L-glutathione reduced (≥98%) and 1-butanethiol (98%) were obtained from Alfa Aesar. Tetraoctylammonium bromide, TOABr (>98%), thiourea (>99%) and *(S)*-(-)-2-methyl-1-butanol (>98.0%) were ordered from TCI Chemicals. 2-phenylethanethiol (98%), chloroform (99.0 – 99.4%) and *trans*-2-[3-(4-*tert*-butylphenyl)-2-methyl-2-propenyliden]-malononitrile (DCTB; ≥99%) were purchased from Sigma Aldrich. Triethylamine (99%) was purchased from Acros Organics. 4-Toluenesulfonyl chloride (98%) was ordered from abcr Gute Chemie. HPLC grade isopropanol, n-hexane and toluene were purchased from Carl Roth. Milli-Q water (resistivity of 18.2 MΩ · cm at 25 °C) was used in the synthesis procedure. All solvents used (dichloromethane, methanol, tetrahydrofuran, toluene, etc.) were at least of synthesis grade and used without further purification. Bio Beads S-X1 support (Bio-Rad) was used for size exclusion chromatography (SEC) and silica gel 60 for column chromatography.

1.2. (S)-2-Methylpropane-1-thiol (2-MeBuSH)

The *(S)*-enantiomer of the chiral thiol ligand used in the nanocluster synthesis was obtained from the corresponding *(S)*-alcohol in a two-step process adapted from Jin and coworkers.[1] To begin with, 7 g (79.5 mmol) *(S)*-2-methylbutan-1-ol and 16.34 g (85.7 mmol) 4-toluenesulfonyl chloride were dissolved in 60 mL of dichloromethane (DCM). Slow addition of 27.49 mL (197.8 mmol) of triethylamine to the ice-cooled mixture resulted in formation of a white precipitate. After stirring at room temperature for 16

h, the white precipitate was removed and the solution was washed with diluted HCl and water. After extraction of the aqueous phase with DCM, the combined organic phases were dried over Na_2SO_4. Removal of DCM by rotary evaporation yielded a clear oil, which was purified by silica column chromatography in 1:3 hexane:EtOAc (EtOAc = ethylacetate). The intermediate (14.50 g, 59.9 mmol) and 4.56 g (59.9 mmol) of thiourea were dissolved in 85 ml of ethanol (EtOH). After refluxing at 80 °C for 72 h, 60 ml of 20% NaOH were added and the reaction mixture kept at 80 °C for another 60 min. The solution was subsequently cooled to room temperature and acidified with 100 ml of 10% HCl. The organic phase was extracted with hexane and dried over Na_2SO_4. Removal of the solvent and impurities was achieved by distillation at 140 °C. The product, which was obtained as a clear oil, was characterized by nuclear magnetic resonance spectroscopy (NMR; see Figures S2-S3).

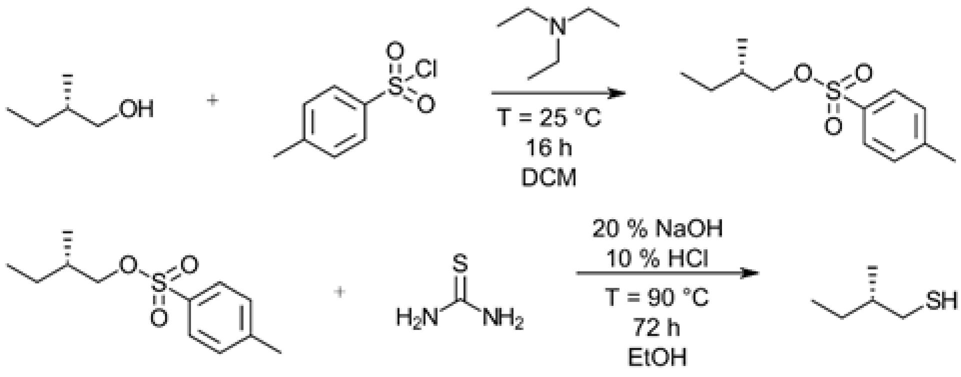

Figure S1. Synthesis of 2-MeBuSH.

1.3. Nanocluster Syntheses

All nanocluster syntheses were performed following modified Brust procedures reported previously.[2-5]

1.3.1 [Au_{25}(2-MeBuS)$_{18}$][TOA] and Au_{25}(2-MeBuS)$_{18}$

[Au_{25}(2-MeBuS)$_{18}$][TOA] and Au_{25}(2-MeBuS)$_{18}$ were synthesized following a protocol by Shihare *et al.*[2] To a 10 mL tetrahydrofuran (THF) solution of 100 mg (0.25 mmol) $HAuCl_4 \cdot 3H_2O$ and 167 mg (0.31 mmol) tetraoctylammonium bromide (TOAB), 156 µL (1.27 mmol) of 2-MeBuSH were added, which resulted in the original orange color of the solution slowly fading out over the course of an hour. The reaction mixture was subsequently reduced by addition of 96 mg (2.54 mmol) $NaBH_4$ in 2 mL ice-cold water. Stirring at room temperature was continued for 2 days, after which the solvent was removed by rotary evaporation and the residue washed with 1:1 H_2O:methanol (MeOH) and purified by size-exclusion chromatography (SEC; THF/Bio-Beads S-X1 support). After elution of a black fraction (which was identified as Au_{144}(2-MeBuS)$_{60}$), a reddish-brown fraction of [Au_{25}(2-MeBuS)$_{18}$][TOA] and a greenish fraction containing Au_{25}(2-MeBuS)$_{18}$ could be isolated. Approximate yields of 25% ([Au_{25}(2-MeBuS)$_{18}$][TOA]), 6% (Au_{25}(2-MeBuS)$_{18}$) and 8% (Au_{144}(S-MeBu)$_{60}$) were obtained.

1.3.2 [Au_{25}(S-Bu)$_{18}$][TOA] and Au_{25}(S-Bu)$_{18}$

The synthesis protocol was identical to the one described in Section 1.3.1, except for the addition of 136 µl (1.26 mmol) of butanethiol (SH-Bu) instead of 2-MeBuSH. No Au_{144}(S-Bu)$_{60}$ was isolated in this synthesis and the respective approximate yields of [Au_{25}(S-Bu)$_{18}$][TOA] and Au_{25}(S-Bu)$_{18}$ were 23% and 4%.

1.3.3 Au_{38}(2-MeBuS)$_{24}$

Au_{38}(2-MeBuS)$_{24}$ was synthesized by adapting the protocol reported by Stellwagen and co-workers.[3] 50 mg (0.15 mmol) $HAuCl_4 \cdot 3H_2O$ and 155 mg (0.5 mmol) of *L*-glutathione (GSH) were dissolved in 8 mL MeOH and 3.5 mL H_2O, yielding a white suspension. After cooling the mixture to 0 °C, 47 mg (1.2

mmol) $NaBH_4$ suspended in 2.4 mL ice-cold water were added, which resulted in the formation of a black precipitate. The reaction was stirred at 0 °C for 1 h. After separating the black precipitate by centrifugation, the solid was dissolved in 2.4 mL H_2O, 1.5 mL acetone and 2 mL (15.6 mmol) 2-MeBuSH. The reaction was continued at 80 °C for 16 h. The phases were separated and the aqueous phase washed with DCM. The organic phase was dried and the resulting black precipitate washed with ethanol for purification (yield with respect to $HAuCl_4{\cdot}3H_2O$ was 78%).

1.3.4 Au_{38}(2-PET)$_{24}$

Au_{38}(2-PET)$_{24}$ was prepared by following the procedure published by Pollitt and co-workers.[4] First, 1 g (2.9 mmol) $HAuCl_4{\cdot}3H_2O$ and 3.17 g (10.3 mmol) GSH were dissolved in 100 ml acetone, giving a yellow suspension, which was stirred at 0 °C for 30 min. Next, an ice-cold solution of 30 ml H_2O and 0.98 g (25.9 mmol) $NaBH_4$ was poured in carefully, resulting in the formation of a black precipitate. After decanting the solvent and drying the solid, 6 ml EtOH, 10 ml toluene, 30 ml water and 10 ml (74.7 mmol) 2-PET were added. The mixture was stirred at 80 °C for 4 h and subsequently cooled to room temperature. 50 ml hexane were added and the black precipitate removed by filtration. After washing several times with MeOH, the crude product was redissolved in DCM and dried by rotary evaporation at 30 °C. For purification of the crude product, SEC (THF, Bio-Beads SX-1 support) was performed (yield of final product was 6% with respect to $HAuCl_4{\cdot}3H_2O$).

1.3.5 Au_{38}(S-Bu)$_{24}$

The synthesis was performed in an analogous fashion to the procedure presented in Section 1.3.3, with the difference that 20 ml (185.6 mmol) of SH-Bu were added instead of the 2-MeBuSH. Furthermore, after stirring at 80 °C for 16 h, the cluster was precipitated with 88 ml of a 1:10 H_2O:MeOH solution. The black/violet solid was then filtered, washed with EtOH, and subsequently extracted with toluene and dried. Yield with respect to $HAuCl_4{\cdot}3H_2O$ was 26%.

1.3.6 Au_{144}(2-MeBuS)$_{60}$

Au_{144}(2-MeBuS)$_{60}$ nanoclusters were prepared by modifying the procedure presented by Qian *et al.*[5] 236 mg (0.6 mmol) $HAuCl_4{\cdot}3H_2O$ were mixed with 380 mg (0.7 mmol) TOAB and dissolved in 30 mL MeOH. The red solution was stirred for 15 minutes at room temperature, after which 394 µL (3.2 mmol) 2-MeBuSH were added, giving a white suspension. After stirring for 15 min at room temperature, the polymer suspension was reduced using a cooled solution of 227 mg (6 mmol) $NaBH_4$ dissolved in 12 mL water, yielding a black precipitate. The black solution was stirred for another 5 h at room temperature. Subsequently, the black precipitate was separated by centrifugation and washed several times with methanol. The crude product was purified by SEC (THF, Bio-Beads S-X1 support). Au_{144}(2-MeBuS)$_{60}$ eluted as the first fraction (black), followed by another black fraction (identified as Au_{38}(2-MeBuS)$_{24}$) and Au_{25}(2-MeBuS)$_{18}$ in anionic (reddish-brown) and neutral (greenish) charge state. The yield of Au_{144}(2-MeBuS)$_{60}$ with respect to $HAuCl_4{\cdot}3H_2O$ was 73%.

1.3.7 Au_{144}(S-Bu)$_{60}$

The synthesis protocol was analogous to the one described in Section 1.3.6, except for the addition of 343 µl (3.2 mmol) HS-Bu instead of 2-MeBuSH. The yield of Au_{144}(S-Bu)$_{60}$ with respect to $HAuCl_4{\cdot}3H_2O$ was 38%.

2 NMR Spectra of the 2-MeBuSH Ligand

Nuclear magnetic resonance (NMR) spectroscopy was measured on a Bruker Avance 400 MHz NMR spectrometer. Samples were dissolved in $CDCl_3$ and the solvent signal was used as internal reference. Chemical shifts relative to trimethylsilane (TMS) are reported.

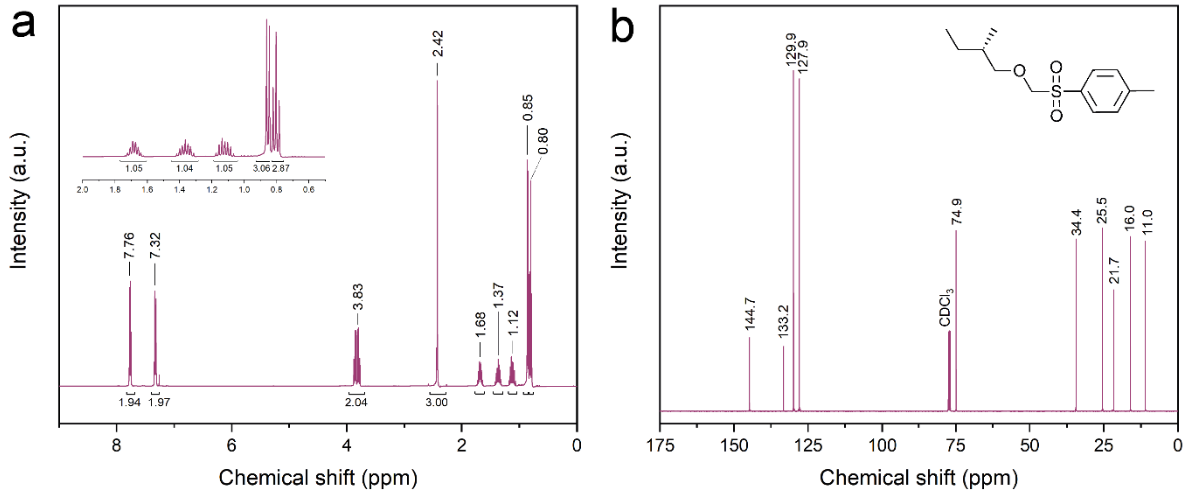

Figure S2. ^{1}H (a) and ^{13}C-NMR spectrum (b) of (S)-2-methylbutyl 4-methylbenzensulfonate.

^{1}H-NMR (400 MHz, $CDCl_3$, TMS): d = 0.80 (t, 3H, CH_2CH_3); 0.85 (d, 3H, $CHCH_3$); 1.12 (m, 1H, $CHCH_2CH_3$); 1.37 (m, 1H, $CHCH_2CH_3$); 1.68 (m, 1H, CH); 2.42 (s, 3H, Ar-CH_3); 3.83 (m, 2H, O-CH_2); 7.32 (d, 2H, Ar-H); 7.76 (d, 2H, Ar-H).

^{13}C ^{1}HNMR (101 MHz, $CDCl_3$, TMS): d = 11.0 (s); 16.0 (s); 21.7 (s); 25.5 (s); 34.4 (s); 74.9 (s); 127.9 (s); 129.9 (s); 133.2 (s); 144.7 (s).

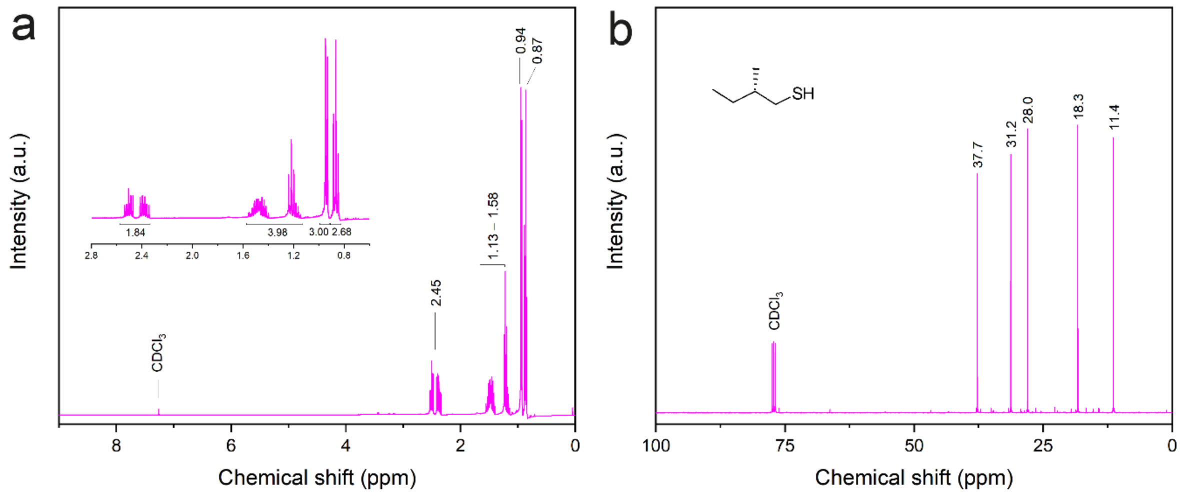

Figure S3. ^{1}H (a) and ^{13}C-NMR spectrum (b) of (S)-2-methylbutanethiol.

^{1}H-NMR (400 MHz, $CDCl_3$, TMS): d = 0.87 (t, 3H, CH_2CH_3); 0.94 (d, 3H, $CHCH_3$); 1.13–1.58 (m, 4H, $CHCH_2CH_3$, SH); 2.45 (m, 2H, S-CH_2).

^{13}C ^{1}HNMR (101 MHz, $CDCl_3$, TMS): d = 11.4 (s); 18.3 (s); 28.0 (s); 31.2 (s); 37.7 (s).

3 Ultraviolet–Visible Spectroscopy

UV-Vis spectroscopy was performed on a UV-1600PC spectrometer using cuvettes of 1 cm pathlength. Different solvents (DCM, toluene, THF) were used to dissolve the Au nanoclusters depending on the specific reaction step.

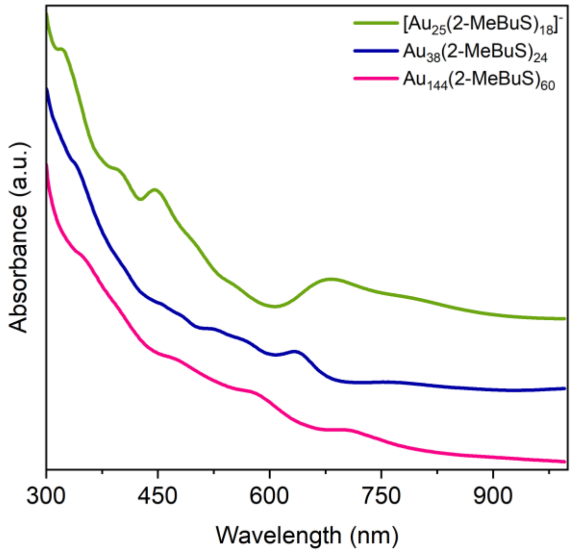

Figure S4. Representative UV-Vis spectra of the synthesized nanoclusters.

4 Matrix-assisted Laser Desorption/Ionization (MALDI-MS) Spectra of the Au Nanoclusters

Matrix-assisted laser desorption/ionization mass spectrometry (MALDI-MS) was conducted on a Bruker Ultraflextreme MALDI-TOF instrument equipped with a Nd:YAG laser in linear mode. Each spectrum was obtained by averaging 5000 single shots (split in packets of 500 shots). Spectra were obtained at 10% (Au_{25} and Au_{38}) or 30% (Au_{144}) laser power. *trans*-2-[3-(4-*tert*-Butylphenyl)-2-methyl-2-propenyliden]-malononitrile (DCTB) was used as matrix. Sample and matrix solutions were prepared in toluene.

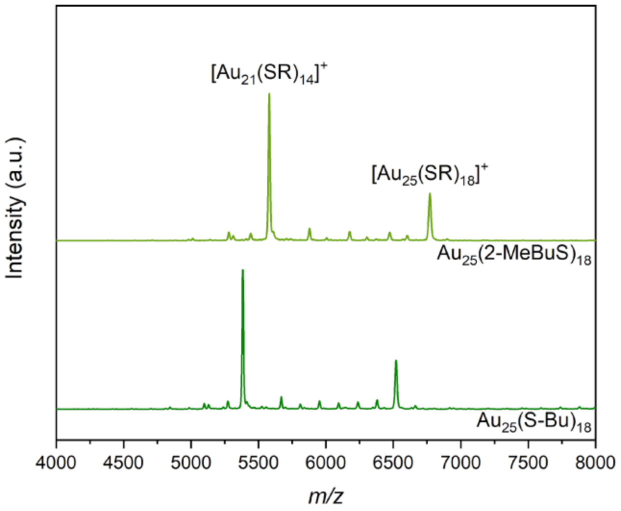

Figure S5. MALDI-MS spectra of $[Au_{25}(2\text{-MeBuS})_{18}]^-$ (top) and $[Au_{25}(S\text{-Bu})_{18}]^-$ (bottom). Note that the counterion cannot be identified due to measuring in positive mode.

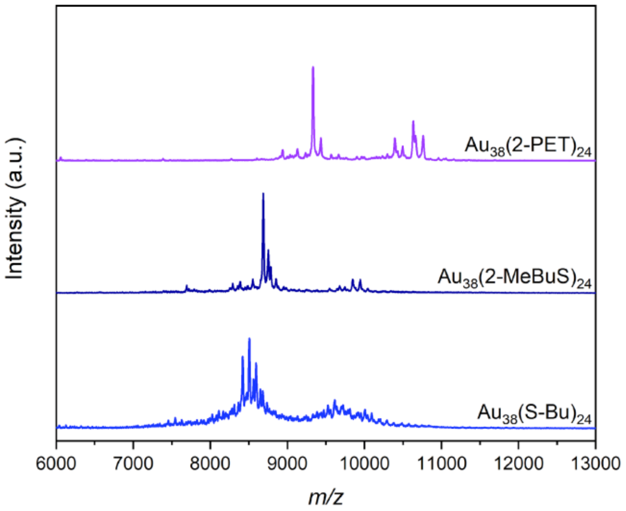

Figure S6. MALDI-MS spectra of $Au_{38}(2\text{-PET})_{24}$ (top), $Au_{38}(2\text{-MeBuS})_{24}$ (middle) and $Au_{38}(S\text{-Bu})_{24}$ (bottom).

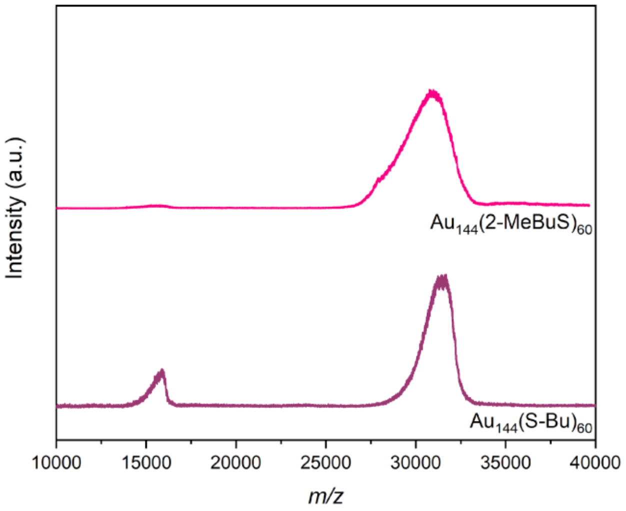

Figure S7. MALDI-MS spectra of $Au_{144}(2\text{-}MeBuS)_{60}$ (top) and $Au_{144}(S\text{-}Bu)_{60}$ (bottom).

5 Additional Circular Dichroism (CD) Spectra

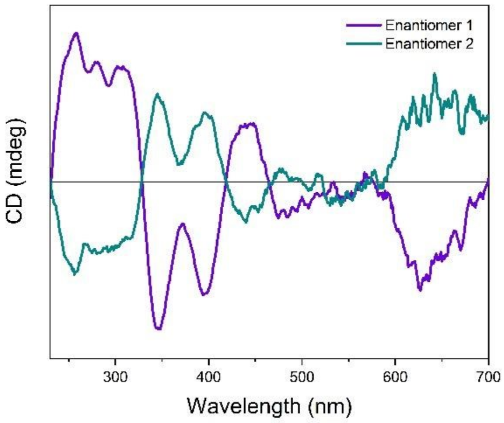

Figure S8. CD spectra of two enantiomers of $Au_{38}(2\text{-}PET)_{24}$

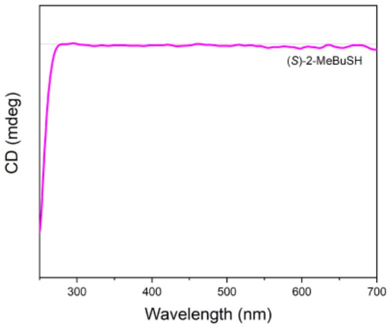

Figure S9. CD spectrum of the (S)-2-methylbutanethiol ligand.

6 HPLC Separation of Au_{38} Nanoclusters

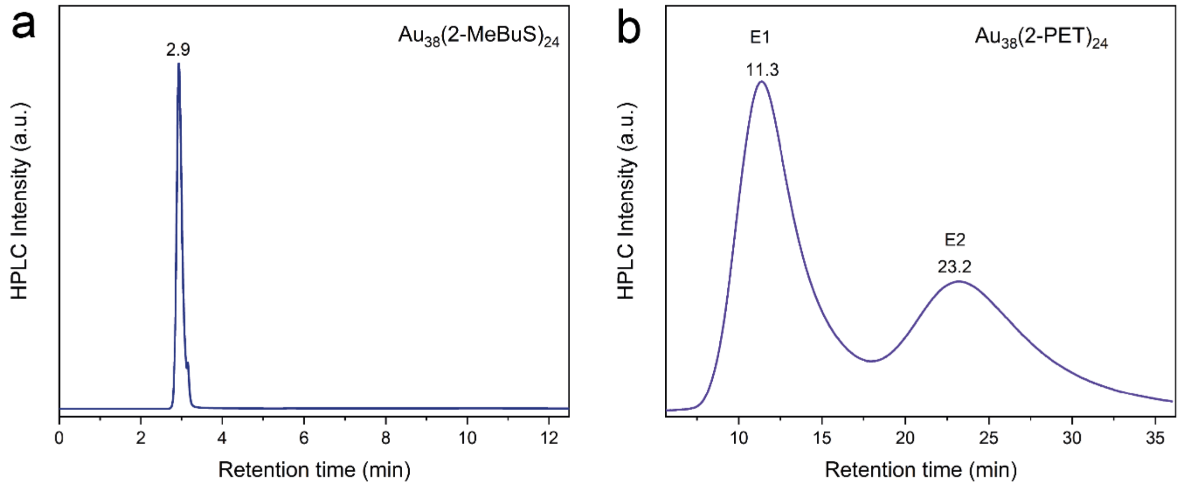

Figure S10. HPLC chromatograms of $Au_{38}(2\text{-MeBuS})_{24}$ (a) and $Au_{38}(2\text{-PET})_{24}$ (b)

7 Computational Methods

7.1 Model Structure and Properties of Au_{25} and Au_{38} Clusters

For the DFT calculations of Au_{25} and Au_{38} clusters, the electronic structure calculations were carried out using the Amsterdam Modeling Suite (AMS) 2021.1 package.[6] The initial structures before optimization were based on previously reported structures[7-9] and the ligands were replaced with a gas-phase optimized structure of 2-MeBuSH. All geometry optimizations of the clusters were done in the gas phase, using a generalized gradient approximation (GGA) Becke Perdew exchange-correlation functional (BP86).[10-11] A double zeta polarized basis set (DZP) was used.[12] The zeroth-order regular approximation (ZORA) was used to account for scalar relativistic effects.[13-14] The gradient convergence was set to 10^{-3} and the energy convergence to 10^{-4}. For Au_{38}, after a first optimization, further isomers of each structure were created by either editing the ligand positions using the MacMolPlt[15] software or by sampling structures from classical molecular dynamics simulations (see details in section 6.3). Geometry optimizations were performed on all newly created structures.

Optical absorption and circular dichroism (CD) spectra were obtained after a linear response TD-DFT+TB[16] calculation and subsequent convolution of the excitation energies into a spectrum by applying a Gaussian fit with a full width half-maximum (FWHM) of 30 nm (15 nm only for the pure *(S)*-MeBuSH ligand in Figure S28). TD-DFT+TB is an approximate TD-DFT method that builds a tight-binding-like excited state calculation on a standard DFT ground state calculation; this method does not require tight-binding parameters, but greatly reduces the required computational time. All TD-DFT+TB calculations were performed at the BP86/DZP level of theory. The CD spectrum of the *(S)*-2-MeBuSH ligand was obtained by employing a Gaussian fit with a FWHM of 15 nm. For the fitting procedure, please see refs.[17-18] The calculated excitation energies were converted to wavelength units (nm) prior to fitting in order to compare with the experimental spectra. To confirm that TD-DFT+TB was applicable for the calculations of the nanoclusters in question, some spectra were also simulated employing time dependent-density functional theory (TD-DFT) (see Figure S13).[19]

7.2 Model structure and properties of Au_{144}(2-MeBuS)$_{60}$ cluster

The electronic, optical, and chiral properties of the Au_{144}(2-MeBuS)$_{60}$ cluster were calculated using DFT as implemented in the software GPAW.[20] GPAW uses real-space grids and scalar-relativistic corrections for metal atom setups. As a starting structure for modeling, we used the crystal structure of $Au_{144}(SCH_2Ph)_{60}$ cluster[21] and replaced the original SCH_2Ph with 2-MeBuS ligands. We selected the right-handed enantiomer of the cluster and the corresponding chirality of the ligand. The symmetry of the cluster structure regarding the metal core, metal-ligand interface, and the bonding directions of the ligands within the protecting SR-Au-SR units was fixed during replacement. At first, the symmetrical model structure was optimized using the Perdew-Burke-Ernzerhof (PBE) xc-functional[22] and a 0.2 Å grid spacing. Optimization was continued until the maximum forces acting on atoms were below 0.05 eV/Å. The electronic structure was analyzed by projecting the density of states to spherical harmonic functions centered at the center of the mass of the cluster with a cutoff radius of 15.0 Å.[23] The results of the analysis were shown together with analyzing the origin of the chirality. Optical absorption and CD spectra were calculated using linear response time-dependent DFT and PBE as a kernel.[24] Spectra were calculated using a 0.3 Å grid spacing for better computational efficiency. The origins of the CD spectrum features were analyzed using rotatory transition contribution maps, which show the negative and positive contributions of the individual electron-hole transitions to the total rotatory strength as decomposed into the Kohn-Sham basis.[25-26] The analysis was done just in one direction, along the main principal axis of moments of inertia of the cluster, which is adequate for this nearly spherical system.

7.3 Molecular dynamics simulations and essential dynamics analysis

For the thiolate-protected gold clusters, molecular dynamics (MD) simulations were carried out using the GROMACS package.[27-29] We use an AMBER-compatible molecular mechanics force field for the thiolate protected gold clusters.[30-31] For all MD simulations, periodic boundary conditions and the minimum image convention were employed. The ‘v-rescale’ thermostat was used to control the temperature with a time constant of 0.1 ps.[32] For the NPT simulations, isotropic pressure coupling with a reference pressure of 1 bar was achieved using the Berendsen barostat with a time constant of 1.0 ps.[33] Prior to the production MD simulations, the energy of the solvated system was minimized using the steepest descent method followed by a short equilibration consisting of 10 ns NVT (constant number of particles, volume, and temperature) at 200 K followed by 10 ns NPT (constant number of particles, pressure, and temperature) at 298.15 K and 1 bar pressure using the v-rescale thermostat and Berendsen barostat.[33] During the equilibrations, the heavy atoms of the nanocluster were position-restrained using a harmonic potential with a force constant of 1000 kJ/mol/nm^2 to allow the solvent to relax around the nanocluster. For improved performance, bonds involving hydrogens were constrained using the LINear Constraint Solver (LINCS) algorithm.[34] The use of bond constraints allowed for a 2-fs time step to be used for the integration of the equations of motion, which was performed using the Leap-Frog[35] algorithm. The PME technique[36] was used to calculate electrostatic interactions with a cutoff distance of 1.0 nm for the real space contributions, cubic interpolation, a maximum fast Fourier transform grid spacing of 0.12 nm for the reciprocal space sum, and tinfoil boundary conditions. Lennard-Jones interactions were cut-off at 1.0 nm and long-range dispersion corrections were applied for the energy and pressure. The Verlet cut-off scheme[37] was used, with the allowed energy error due to the Verlet buffer set to the Gromacs default of 0.005 kJ/mol/ps/atom.

For the Au_{38} cluster, we chose two lower energy isomers as the starting structure to perform classical molecular dynamics (MD) simulations, and GAFF[38] was applied for dichloromethane molecules. The gold thiolate nanocluster system was simulated in a cubic simulation box (side length = 10 nm) with 8234 dichloromethane solvent molecules. Production simulations were carried out for a total of 10 ns with the NPT ensemble at 298.15 K and 1 bar, continuing from the equilibrated structures and velocities. Structures were extracted every 1 ns from both trajectories.

For the Au_{144} cluster, the DFT-optimized model of the Au_{144}(2-MeBuS)$_{60}$ cluster was solvated in a periodic cubic box of methanol (997 nm^3). 300 ns of production MD was carried out by keeping the temperature at 300 K with the velocity-rescale thermostat[39] and pressure at 1 bar using Parinello-Rahman barostat[40] with a period of 2.0 ps. Then, the MD trajectory was analyzed with so-called essential dynamics (ED).[41-42] ED analysis represents the principal motion directions of the system. By using a covariance matrix constructed from atomic coordinates, the most important elements of the position fluctuations in the MD trajectory are extracted (eigenvalue-eigenvector decomposition). Thus, we used this analysis to find out the most relevant structural conformations of the cluster and their basins seen during the MD simulation. We selected one representative snapshot structure (MD-frame 3555) around the observed minimum regions of the free energy landscape and compared its properties with the symmetrical model structure. Therefore, we repeated the calculations of the optical and CD spectra for the optimized snapshot structure the same way as for the symmetrical model structure. In addition to ED analysis, we studied the behavior of the radius of gyration (Rg) during the MD simulation using VMD software[43] and analyzed the flipping of the ligands within the protecting units. Ligand conformations were analyzed using the dihedral angle between the vectors defined along the S-C bond at both ends of each unit. In practice, 0 degrees means ligands pointing to the same side of the unit, while 100 and -100 degrees mean ligands pointing to different sides of the unit Possible angles are restricted by spatial effects on the cluster surface which makes the angle range deviate from the ideal from +180 to -180 degrees.

8 Additional Information and Results for the Calculations of Au_{38}(2-MeBuS)$_{24}$

In the following section, isomer 1 corresponds to structures obtained after modification of the experimental Au_{38} crystal structure,[7] whereas isomer 2 was obtained starting from the lowest energy structure found by Lopez-Acevedo *et al.* (denoted as *JACS2010* structure).[8] The appendix *a* corresponds to the anti-clockwise and the appendix *b* to the clockwise enantiomer.

Table S1: Relative energies of the lowest energy calculated Au_{38}(2-MeBuS)$_{24}$ isomers. A=anti-clockwise and C=clockwise staple rotation. Isomers 1 are crystal structure[7] based and isomers 2 were obtained starting from the calculated structure by Lopez-Acevedo *et al.*[8]

Cluster isomer	Structure	Relative Energy (kJ/mol)	
		enantiomer	overall
A	isomer 1a	46.7	46.7
**A**	**isomer 2a**	**0**	**0**
C	isomer 1b	7.7	7.9
C	isomer 2b	**0**	0.2

Figure S11 shows a comparison of the two different anti-clockwise isomers 1a and 2a. For isomer 1a, the ligands in the monomeric staple units (see side view in (a)) are mostly facing outwards, whereas the ones of isomer 2a are also a slightly tilted up- and downwards, respectively. This is due to the different orientation of the -S(R)–Au–S(R)- units, which restricts the orientation of the hydrocarbon framework of the 2-MeBuSH ligand in isomer 1a. Furthermore, the top view of the nine ligands in the upper dimeric staple units show that isomer 2a has a very symmetric arrangement of these nine ligands, with each subset (i.e. the three top, middle and bottom ligands in line of sight) mostly following the idealized D_3 symmetry of the cluster. For isomer 1a, the same nine ligands are arranged in a much less symmetric fashion. Whereas the bottom three ligands still take symmetrical positions with respect to each other, the topmost ones as well as the ones in the middle of the staple units do not. This implies that the symmetry of isomer 1a is reduced as compared to 2a, which might be related with higher energy. However, besides symmetry, other structural factors (for example the different arrangement of the monomeric staples and its implication for the orientation of the surrounding ligands) will affect that as well.

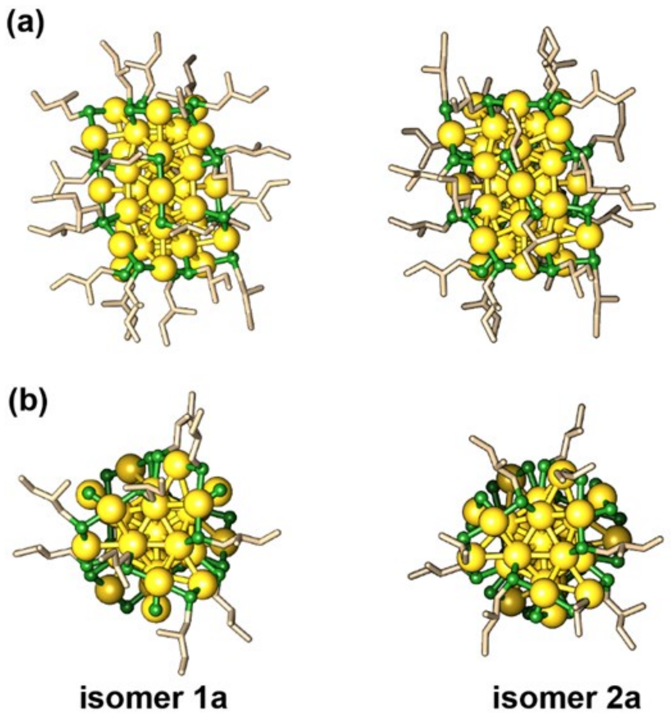

Figure S11: Structures of isomer 1a and 2a: (a) side view and (b) top view. Note that in (b), the hydrocarbon framework of all but the 9 ligands in the dimeric staples on top is not shown to allow for better visualization.

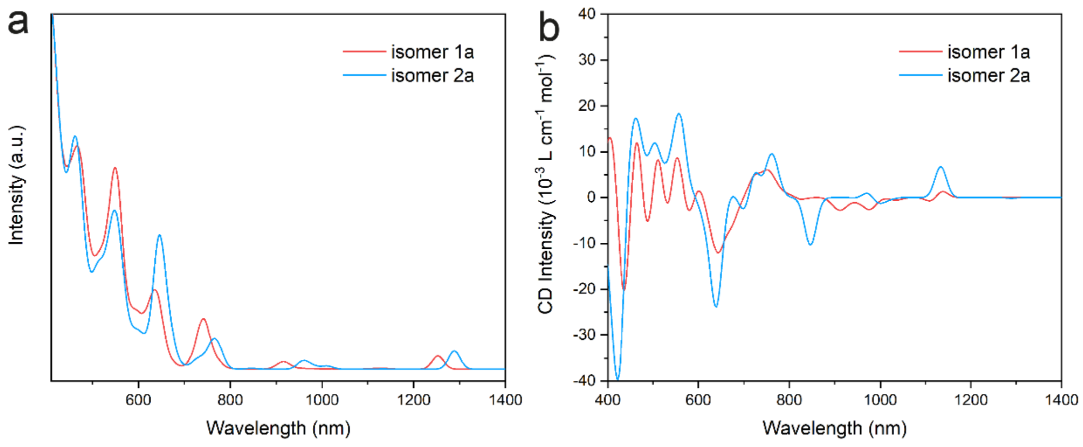

Figure S12: Theoretical UV-Vis (a) and CD spectra (b) of isomers 1a (crystal structure) and 2a (*JACS2010*) in gas phase.

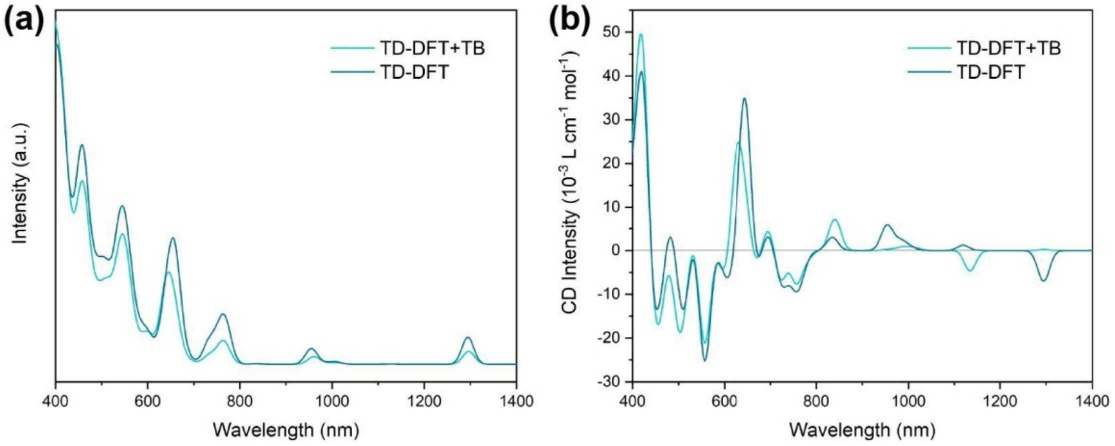

Figure S13: Comparison of the UV-Vis (a) and CD spectra (b) of Au_{38}(2-MeBuS)$_{24}$ isomer 2b obtained by TD-DFT and TD-DFT+TB.

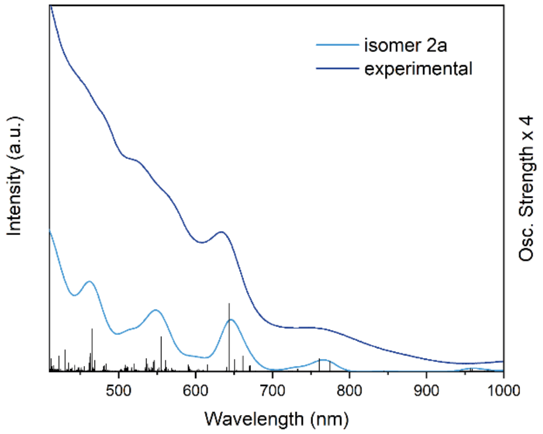

Figure S14: Experimental UV-Vis spectrum of Au_{38}(2-MeBuS)$_{24}$ and theoretical optical absorption spectrum including oscillator strength of isomer 2a in gas phase. Note that the experimental UV-Vis spectrum is offset on the intensity axis from the theoretical one to allow for better visualization.

Table S2. Positions of the features A-H in the measured and in the calculated CD spectra as labeled in the Figure 4.

Labeled feature	Calculated position	Measured position
A	762 nm	≈ 775 nm
B	726 nm	745 nm
C	698 nm	not assigned
D	639 nm	641 nm
E	557 nm	≈ 555 nm
F	504 nm	450 nm
G	462 nm	433 nm
H	422 nm	399 nm

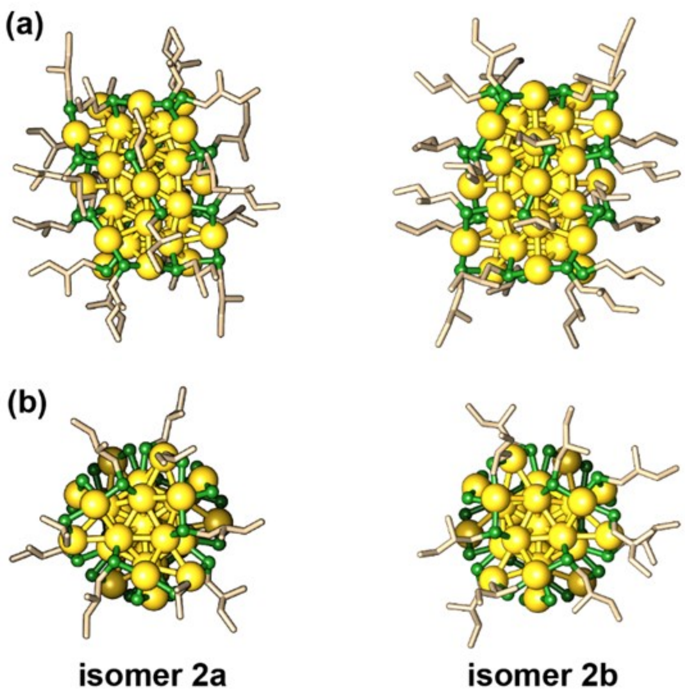

Figure S15: Structures of isomer 2a and 2b: (a) side view and (b) top view. Note that in (b), the hydrocarbon framework of all but the 9 ligands in the dimeric staples on top is not shown to allow for better visualization.

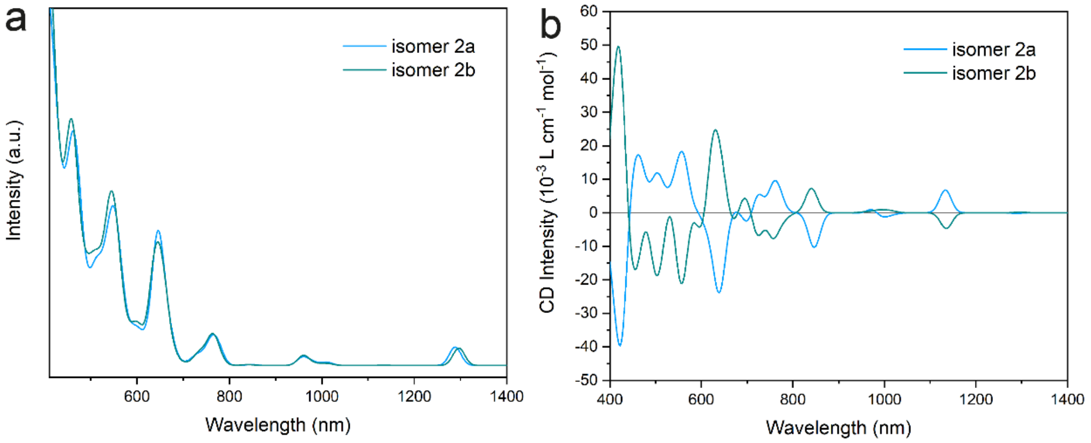

Figure S16: Theoretical UV-Vis (a) and CD spectra (b) of isomers 2a and 2b in gas phase.

9 Additional Information and Results for the Calculations of $Au_{144}(2\text{-}MeBuS)_{60}$

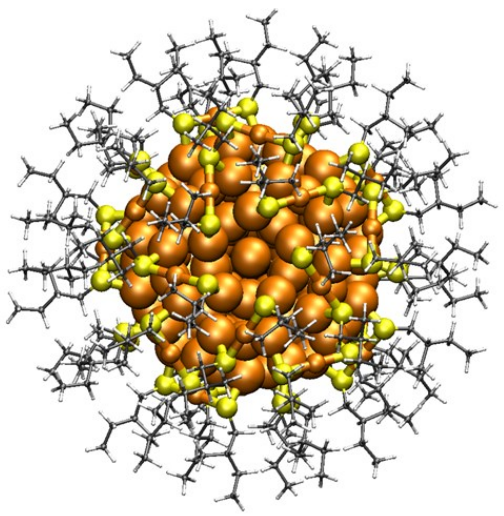

Figure S17. Optimized, symmetrically built, right-handed model structure of $Au_{144}(2\text{-}MeBuS)_{60}$ cluster. Color code: Au, orange; S, yellow; C, gray; H, white.

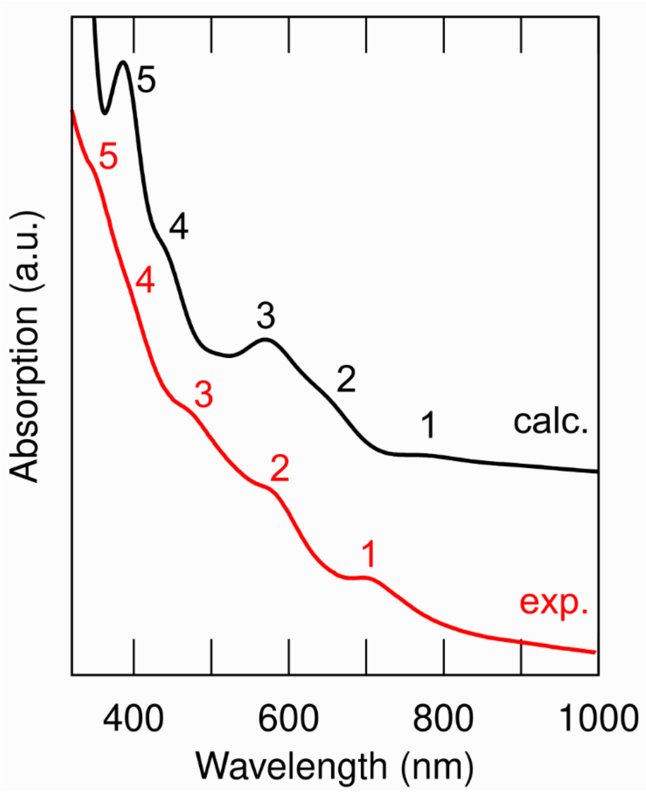

Figure S18. Experimental UV-Vis spectrum of $Au_{144}(2\text{-}MeBuS)_{60}$ cluster (red curve) compared to calculated optical absorption spectrum of the symmetrically built model cluster shown in Figure S17 (black curve). Observed features are labelled from 1-5.

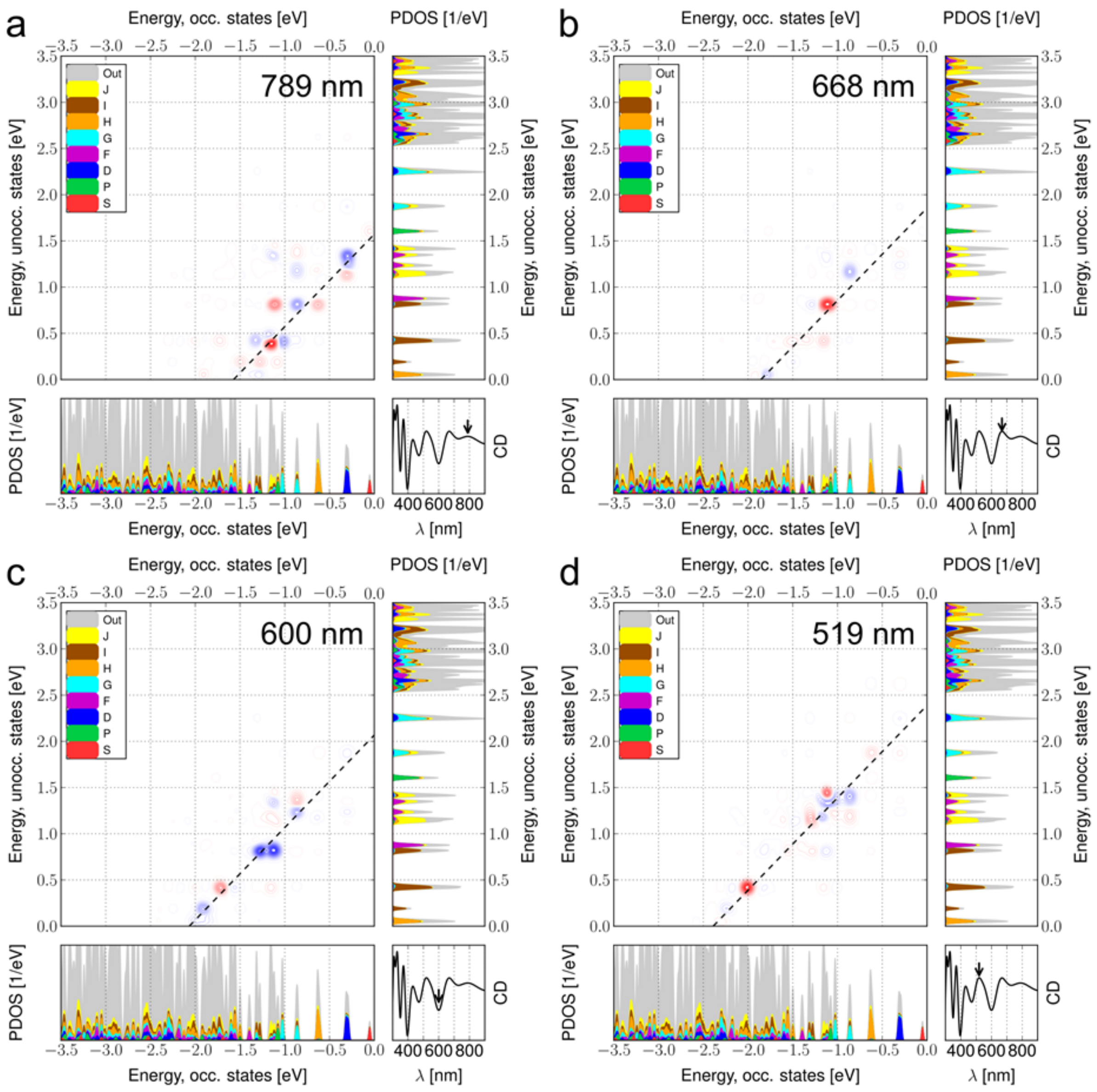

Figure S19. Rotatory strength transition contribution map (RTCM) of the lower energy peaks from A to D. Contour plot shows the negative (blue) and the positive (red) contributions to the total rotatory strength of the system as decomposed to transitions between Kohn-Sham states. Projected density of states to spherical harmonics functions centered at the center of mass of the cluster is shown at the lower left panel for occupied states and at the right upper panel for the unoccupied states. Lower right panel shows the calculated CD spectrum with a small arrow labelling the position of the analyzed peak. Position of the peak is also denoted in wavelength units in the corner of each contour plot panel. Analysis is done with respect to the electric field at the direction of the main principal axis of moments of inertia of the system.

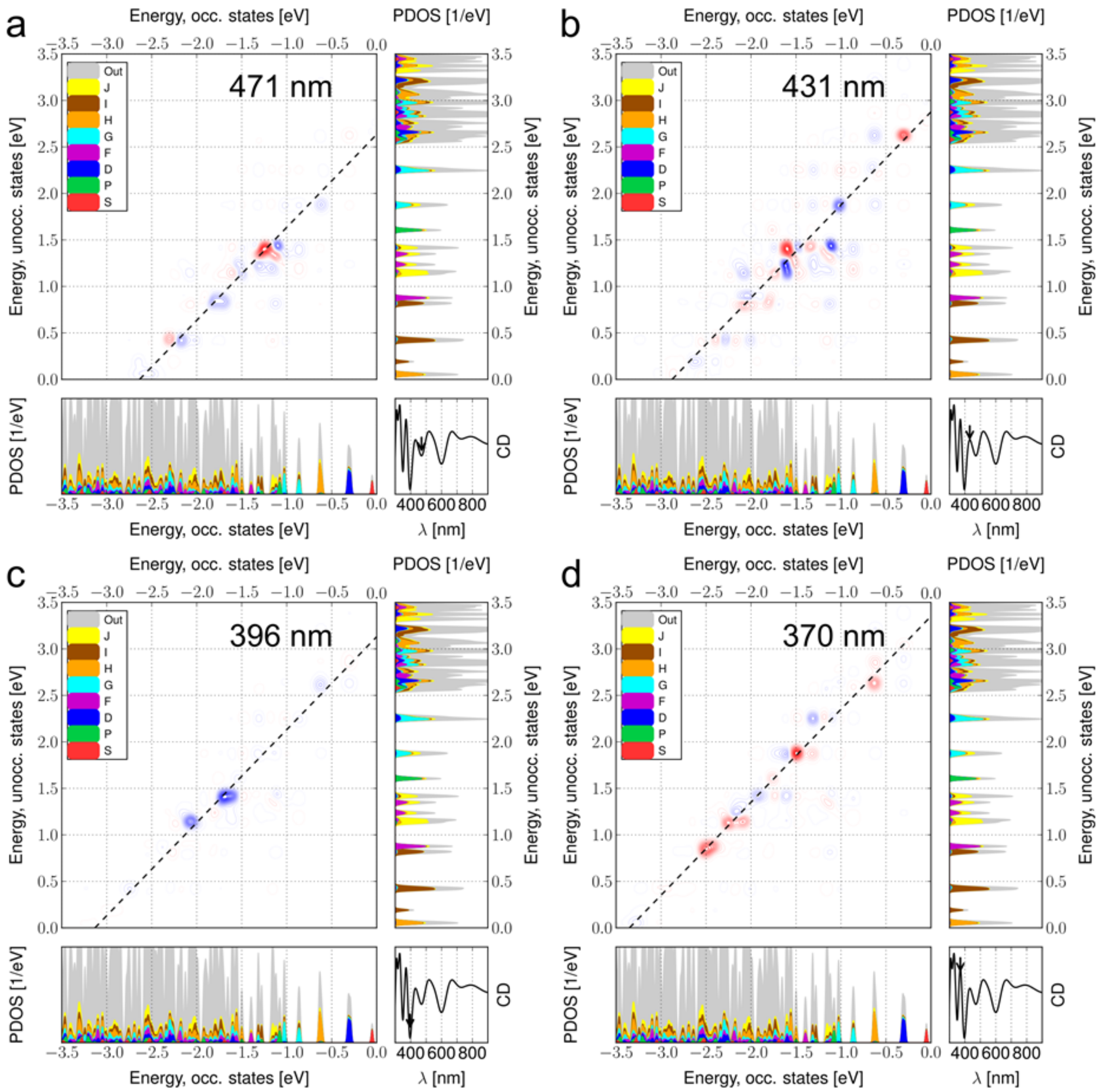

Figure S20. Rotatory strength transition contribution map (RTCM) of the lower energy peaks from E to H.

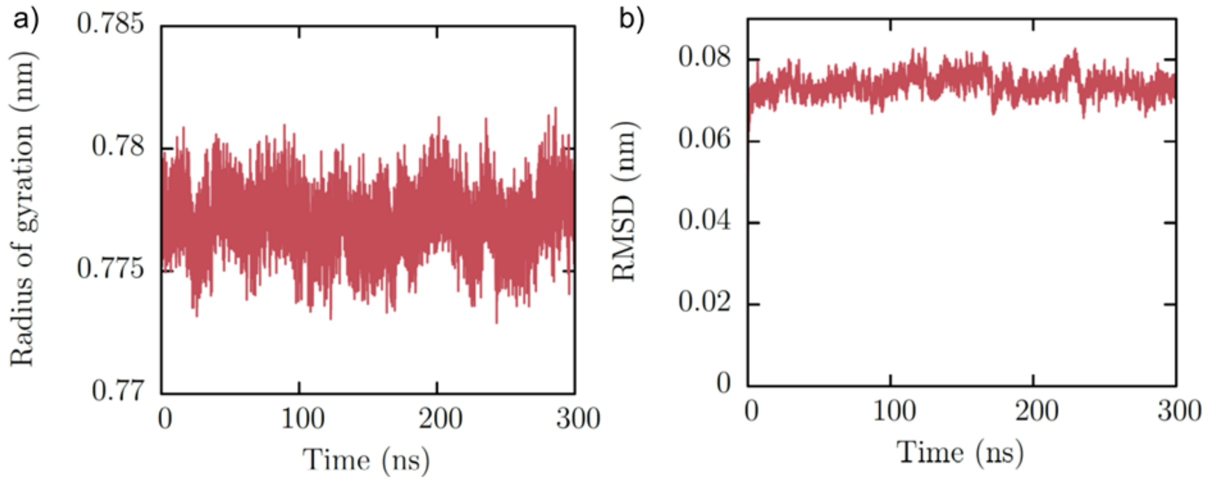

Figure S21. a) Radius of gyration (Rg) of the $Au_{144}(2\text{-}MeBuS)_{60}$ cluster and b) root mean square deviation (RMSD) of atomic positions as a function of simulation time.

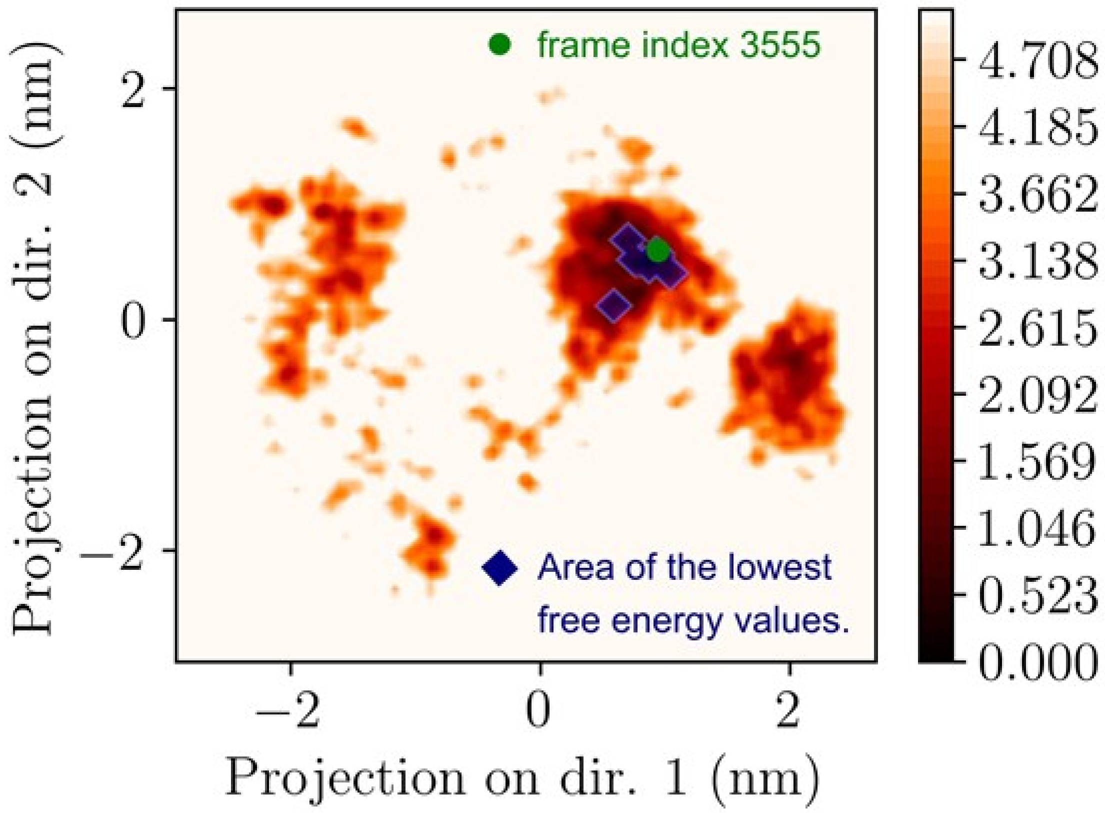

Figure S22. Essential dynamics (ED) analysis showing the conformational free energy for the structures seen during the last 200 ns of 300-ns MD simulation in units of kJ/mol. Projections are made with respect to the two most important principal components given by the analysis. The selected representative snapshot structure is labelled by the green circle, whereas the region for the lowest free energy values is shaded with blue. Zero level of conformational free energy is set to zero, and only the variations below 5 kJ/mol are shown.

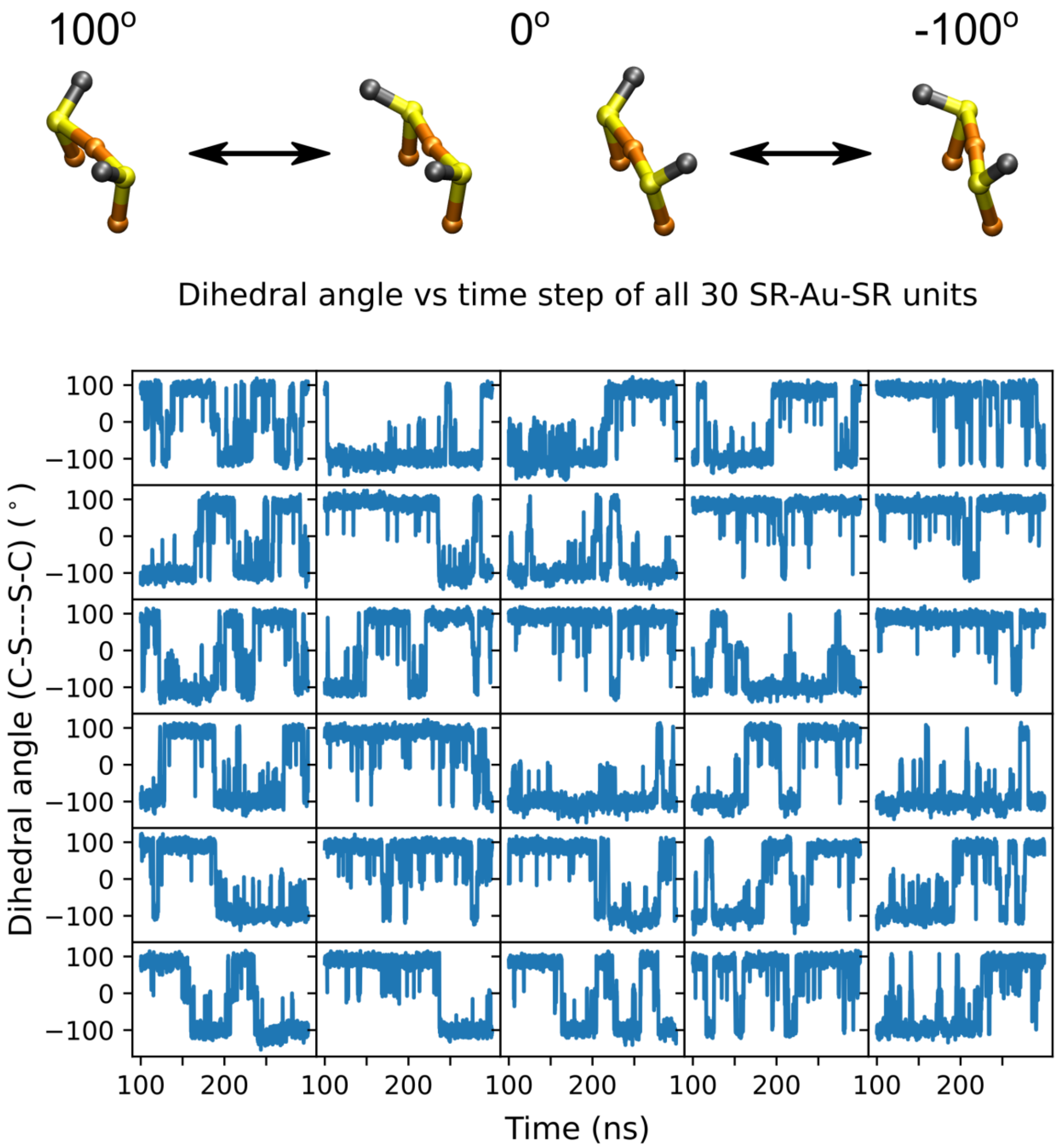

Figure S23. Flipping of ligands within the protecting units from 100 ns to 300 ns during MD simulation determined by the dihedral angle between the S-C bonds at both ends of each protecting unit. Each sub-panel visualizes the conformational fluctuations for one protecting unit. As visualized on top, 0 degrees refers to ligands pointing at the same direction, and 100 and -100 degrees to different direction.

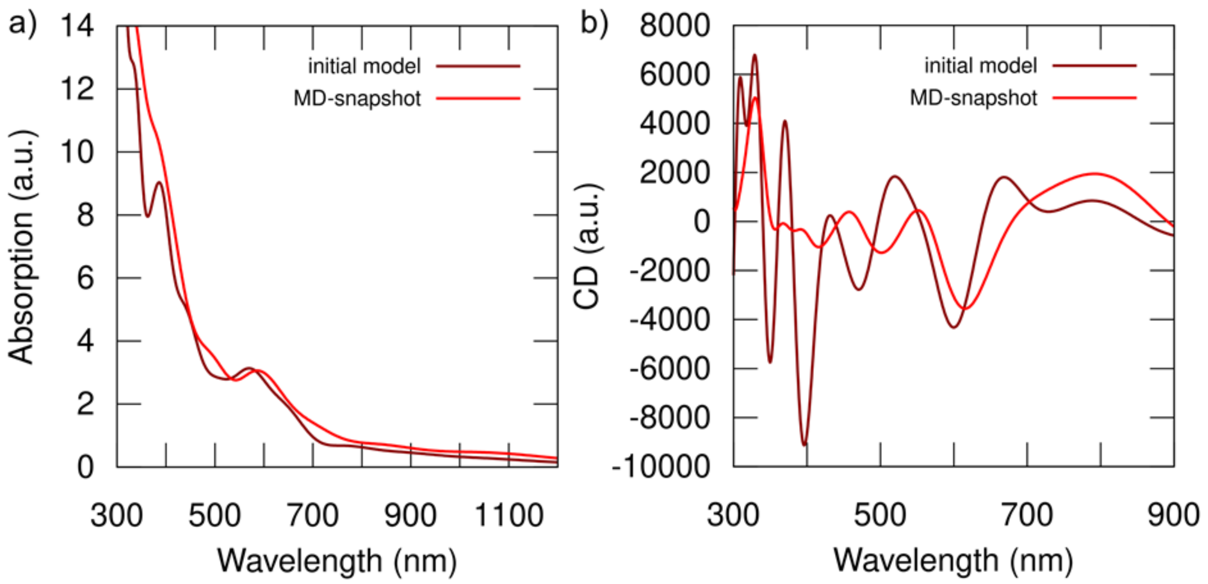

Figure S24. Comparison of the calculated a) optical absorption and b) CD spectra of the symmetrical initial model structure (brown line) and of the MD-snapshot structure (red line).

Table S3. Positions of the features A-H in the measured and in the calculated CD spectra as labelled in the Figure 5.

Labeled feature	Calculated position	Measured position
A	789 nm	770 nm
B	668 nm	744 nm
C	600 nm	673 nm
D	519 nm	532 nm
E	471 nm	460 nm
F	431 nm	427 nm
G	396 nm	388 nm
H	370 nm	348 nm

10 Additional Information and Results for the Calculations of $[Au_{25}(2\text{-}MeBuS)_{18}]^-$

The UV-Vis and CD spectra were also calculated for the intrinsically achiral cluster $[Au_{25}(2\text{-}MeBuS)_{18}]^-$ after optimization at BP86/DZP level of theory. This cluster has an achiral arrangement of its dimeric staple units, thus, its chiral properties are solely due to induction by the chiral *(S)*-2-MeBuSH ligand (see ligand spectra in Figure S28). As can be seen upon comparing to the experimental spectra (Figure S27), the energies of the optical absorption bands are significantly underestimated by TD-DFT+TB at this level of theory. This has been reported for Au_{25} calculated with the BP86 functional before.[44,45] For better visualization, the theoretical spectrum was shifted by +0.5 eV and then shows acceptable agreement with the experimental spectrum, especially considering the shape of the bands. However, for the CD spectra (Figure S27b), significant deviations are noticed as well, which cannot be corrected by a shift of the energy axis only. This shows that the current cluster model is not sufficient for comparison to the experiment. Further refinement, for example at a different level of theory, would be required, but lies outside the scope of this publication.

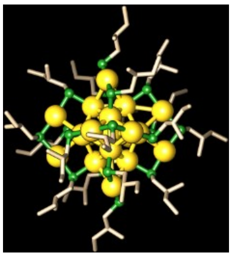

Figure S25: Calculated structure of $[Au_{25}(2\text{-}MeBuS)_{18}]^-$.

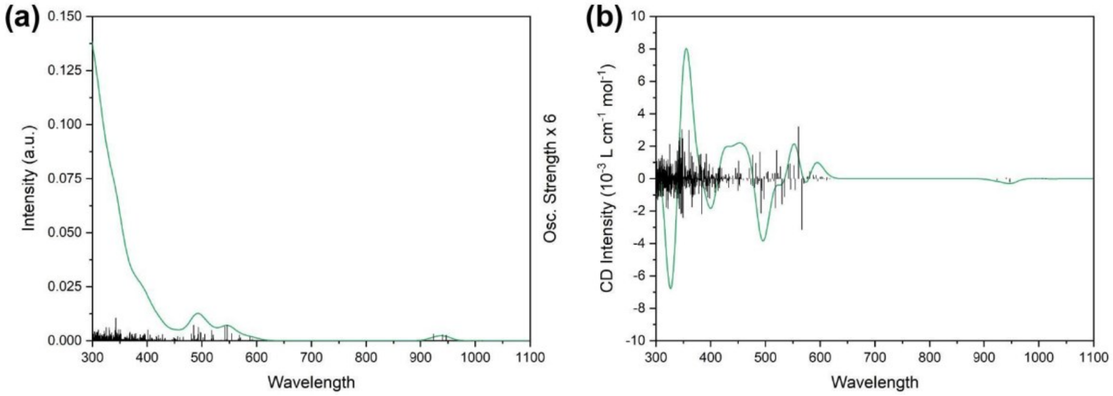

Figure S26: Calculated linear response TD-DFT+TB spectra of $[Au_{25}(2\text{-}MeBuS)_{18}]^-$: (a) oscillator strength and optical absorption spectrum and (b) rotatory strength and CD spectrum.

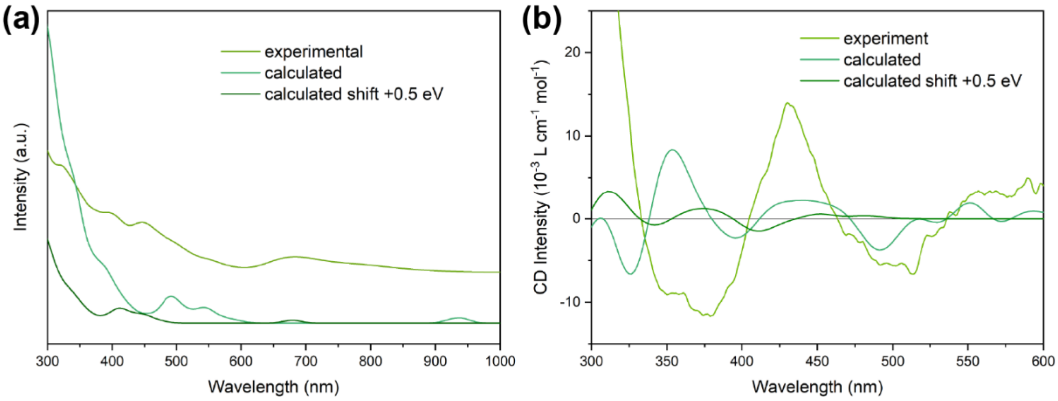

Figure S27: Theoretical spectra of $[Au_{25}(2\text{-}MeBuS)_{18}]^-$ in gas phase and experimental spectrum: (a) UV-Vis and (b) CD spectra. Note that the experimental UV-Vis spectrum is offset on the intensity axis from the theoretical ones to allow for better visualization.

11 Additional Information and Results for the Calculations of *(S)*-2-MeBuSH

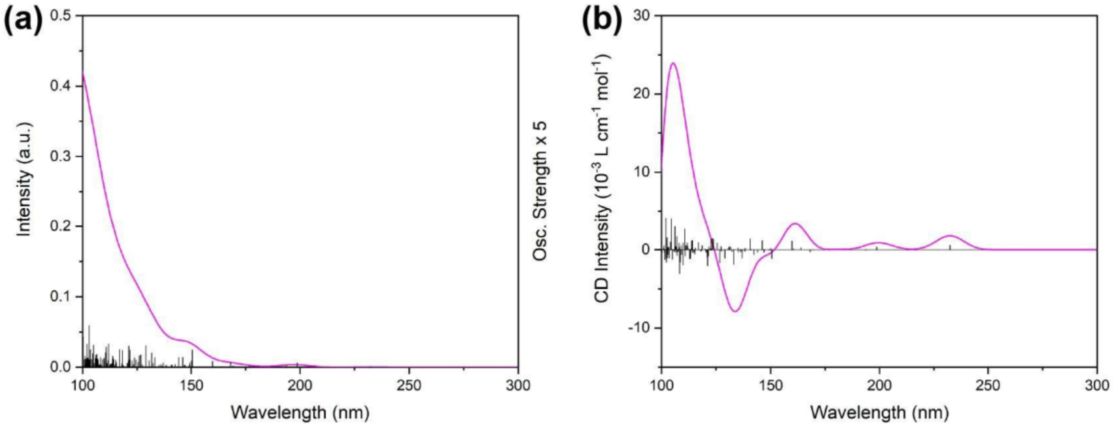

Figure S28: Calculated linear response TD-DFT+TB spectra of *(S)*-2-MeBuSH: (a) oscillator strength and optical absorption spectrum and (b) rotatory strength and CD spectrum.

References

1. Zhu, M.; Qian, H.; Meng, X.; Jin, S.; Wu, Z.; Jin, R., Chiral Au25 Nanospheres and Nanorods: Synthesis and Insight into the Origin of Chirality. *Nano Lett.* **2011,** *11* (9), 3963-3969.
2. Shivhare, A.; Ambrose, S. J.; Zhang, H.; Purves, R. W.; Scott, R. W. J., Stable and recyclable Au25 clusters for the reduction of 4-nitrophenol. *Chem. Commun.* **2013,** *49* (3), 276-278.
3. Stellwagen, D.; Weber, A.; Bovenkamp, G. L.; Jin, R.; Bitter, J. H.; Kumar, C. S. S. R., Ligand control in thiol stabilized Au38 clusters. *RSC Adv.* **2012,** *2* (6), 2276-2283.
4. Pollitt, S.; Truttmann, V.; Haunold, T.; Garcia, C.; Olszewski, W.; Llorca, J.; Barrabes, N.; Rupprechter, G., The Dynamic Structure of Au-38(SR)(24) Nanoclusters Supported on CeO2 upon Pretreatment and CO Oxidation. *ACS Catal.* **2020,** *10* (11), 6144-6148.
5. Qian, H. F.; Jin, R. C., Ambient Synthesis of Au-144(SR)(60) Nanoclusters in Methanol. *Chem. Mater.* **2011,** *23* (8), 2209-2217.
6. te Velde, G.; Bickelhaupt, F. M.; Baerends, E. J.; Fonseca Guerra, C.; van Gisbergen, S. J. A.; Snijders, J. G.; Ziegler, T., Chemistry with ADF. *Journal of Computational Chemistry* **2001,** *22* (9), 931-967.
7. Qian, H.; Eckenhoff, W. T.; Zhu, Y.; Pintauer, T.; Jin, R., Total Structure Determination of Thiolate-Protected Au38 Nanoparticles. *J. Am. Chem. Soc.* **2010,** *132* (24), 8280-8281.

8. Lopez-Acevedo, O.; Tsunoyama, H.; Tsukuda, T.; Hakkinen, H.; Aikens, C. M., Chirality and Electronic Structure of the Thiolate-Protected Au-38 Nanocluster. *J. Am. Chem. Soc.* **2010,** *132* (23), 8210-8218.
9. Heaven, M. W.; Dass, A.; White, P. S.; Holt, K. M.; Murray, R. W., Crystal Structure of the Gold Nanoparticle [N(C8H17)4][Au25(SCH2CH2Ph)18]. *J. Am. Chem. Soc.* **2008,** *130* (12), 3754-3755.
10. Becke, A. D., Density-functional exchange-energy approximation with correct asymptotic behavior. *Physical Review A* **1988,** *38* (6), 3098-3100.
11. Perdew, J. P., Density-functional approximation for the correlation energy of the inhomogeneous electron gas. *Physical Review B* **1986,** *33* (12), 8822-8824.
12. Van Lenthe, E.; Baerends, E. J., Optimized Slater-type basis sets for the elements 1–118. *Journal of Computational Chemistry* **2003,** *24* (9), 1142-1156.
13. Lenthe, E. v.; Baerends, E. J.; Snijders, J. G., Relativistic regular two-component Hamiltonians. *The Journal of Chemical Physics* **1993,** *99* (6), 4597-4610.
14. Lenthe, E. v.; Ehlers, A.; Baerends, E.-J., Geometry optimizations in the zero order regular approximation for relativistic effects. *The Journal of Chemical Physics* **1999,** *110* (18), 8943-8953.
15. Bode, B. M.; Gordon, M. S., Macmolplt: a graphical user interface for GAMESS. *Journal of Molecular Graphics and Modelling* **1998,** *16* (3), 133-138.
16. Rüger, R.; Lenthe, E. v.; Heine, T.; Visscher, L., Tight-binding approximations to time-dependent density functional theory — A fast approach for the calculation of electronically excited states. *The Journal of Chemical Physics* **2016,** *144* (18), 184103.
17. Autschbach, J., Computing chiroptical properties with first-principles theoretical methods: Background and illustrative examples. *Chirality* **2009,** *21* (1E), E116-E152.
18. Karimova, N. V.; Aikens, C. M., Chiroptical Activity in BINAP- and DIOP-Stabilized Octa- and Undecagold Clusters. *J. Phys. Chem. C* **2018,** *122* (20), 11051-11065.
19. van Gisbergen, S. J. A.; Snijders, J. G.; Baerends, E. J., Implementation of time-dependent density functional response equations. *Computer Physics Communications* **1999,** *118* (2), 119-138.
20. Enkovaara, J.; Rostgaard, C.; Mortensen, J. J.; Chen, J.; Dułak, M.; Ferrighi, L.; Gavnholt, J.; Glinsvad, C.; Haikola, V.; Hansen, H. A.; Kristoffersen, H. H.; Kuisma, M.; Larsen, A. H.; Lehtovaara, L.; Ljungberg, M.; Lopez-Acevedo, O.; Moses, P. G.; Ojanen, J.; Olsen, T.; Petzold, V.; Romero, N. A.; Stausholm-Møller, J.; Strange, M.; Tritsaris, G. A.; Vanin, M.; Walter, M.; Hammer, B.; Häkkinen, H.; Madsen, G. K. H.; Nieminen, R. M.; Nørskov, J. K.; Puska, M.; Rantala, T. T.; Schiøtz, J.; Thygesen, K. S.; Jacobsen, K. W., Electronic structure calculations with GPAW: a real-space implementation of the projector augmented-wave method. *Journal of Physics: Condensed Matter* **2010,** *22* (25), 253202.
21. Yan, N.; Xia, N.; Liao, L.; Zhu, M.; Jin, F.; Jin, R.; Wu, Z., Unraveling the long-pursued Au_{144} structure by x-ray crystallography. *Science Advances* **2018,** *4* (10), eaat7259.
22. Perdew, J. P.; Burke, K.; Ernzerhof, M., Generalized Gradient Approximation Made Simple. *Physical Review Letters* **1996,** *77* (18), 3865-3868.
23. Walter, M.; Akola, J.; Lopez-Acevedo, O.; Jadzinsky, P. D.; Calero, G.; Ackerson, C. J.; Whetten, R. L.; Grönbeck, H.; Häkkinen, H., A unified view of ligand-protected gold clusters as superatom complexes. *Proceedings of the National Academy of Sciences* **2008,** *105* (27), 9157-9162.
24. Walter, M.; Häkkinen, H.; Lehtovaara, L.; Puska, M.; Enkovaara, J.; Rostgaard, C.; Mortensen, J. J., Time-dependent density-functional theory in the projector augmented-wave method. *The Journal of Chemical Physics* **2008,** *128* (24), 244101.
25. Deng, G. C.; Malola, S.; Yan, J. Z.; Han, Y. Z.; Yuan, P.; Zhao, C. W.; Yuan, X. T.; Lin, S. C.; Tang, Z. C.; Teo, B. K.; Hakkinen, H.; Zheng, N. F., From Symmetry Breaking to Unraveling the Origin of the Chirality of Ligated Au13Cu2 Nanoclusters. *Angew Chem Int Edit* **2018,** *57* (13), 3421-3425.
26. Malola, S.; Lehtovaara, L.; Enkovaara, J.; Häkkinen, H., Birth of the Localized Surface Plasmon Resonance in Monolayer-Protected Gold Nanoclusters. *ACS Nano* **2013,** *7* (11), 10263-10270.
27. Hess, B.; Kutzner, C.; van der Spoel, D.; Lindahl, E., GROMACS 4: Algorithms for Highly Efficient, Load-Balanced, and Scalable Molecular Simulation. *Journal of Chemical Theory and Computation* **2008,** *4* (3), 435-447.
28. Pronk, S.; Páll, S.; Schulz, R.; Larsson, P.; Bjelkmar, P.; Apostolov, R.; Shirts, M. R.; Smith, J. C.; Kasson, P. M.; van der Spoel, D.; Hess, B.; Lindahl, E., GROMACS 4.5: a high-throughput and highly parallel open source molecular simulation toolkit. *Bioinformatics* **2013,** *29* (7), 845-854.
29. Abraham, M. J.; Murtola, T.; Schulz, R.; Páll, S.; Smith, J. C.; Hess, B.; Lindahl, E., GROMACS: High performance molecular simulations through multi-level parallelism from laptops to supercomputers. *SoftwareX* **2015,** *1-2*, 19-25.

30. Pohjolainen, E.; Chen, X.; Malola, S.; Groenhof, G.; Häkkinen, H., A Unified AMBER-Compatible Molecular Mechanics Force Field for Thiolate-Protected Gold Nanoclusters. *Journal of Chemical Theory and Computation* **2016,** *12* (3), 1342-1350.
31. Lindorff-Larsen, K.; Piana, S.; Palmo, K.; Maragakis, P.; Klepeis, J. L.; Dror, R. O.; Shaw, D. E., Improved side-chain torsion potentials for the Amber ff99SB protein force field. *Proteins: Structure, Function, and Bioinformatics* **2010,** *78* (8), 1950-1958.
32. Bussi, G.; Donadio, D.; Parrinello, M., Canonical sampling through velocity rescaling. *The Journal of Chemical Physics* **2007,** *126* (1), 014101.
33. Berendsen, H. J. C.; Postma, J. P. M.; Gunsteren, W. F. v.; DiNola, A.; Haak, J. R., Molecular dynamics with coupling to an external bath. *The Journal of Chemical Physics* **1984,** *81* (8), 3684-3690.
34. Hess, B.; Bekker, H.; Berendsen, H. J. C.; Fraaije, J. G. E. M., LINCS: A linear constraint solver for molecular simulations. *Journal of Computational Chemistry* **1997,** *18* (12), 1463-1472.
35. Hockney, R. W.; Goel, S. P.; Eastwood, J. W., Quiet high-resolution computer models of a plasma. *Journal of Computational Physics* **1974,** *14* (2), 148-158.
36. Darden, T.; York, D.; Pedersen, L., Particle mesh Ewald: An N·log(N) method for Ewald sums in large systems. *The Journal of Chemical Physics* **1993,** *98* (12), 10089-10092.
37. Páll, S.; Hess, B., A flexible algorithm for calculating pair interactions on SIMD architectures. *Computer Physics Communications* **2013,** *184* (12), 2641-2650.
38. Wang, J.; Wolf, R. M.; Caldwell, J. W.; Kollman, P. A.; Case, D. A., Development and testing of a general amber force field. *Journal of Computational Chemistry* **2004,** *25* (9), 1157-1174.
39. Bussi, G.; Parrinello, M., Stochastic thermostats: comparison of local and global schemes. *Computer Physics Communications* **2008,** *179* (1), 26-29.
40. Parrinello, M.; Rahman, A., Polymorphic transitions in single crystals: A new molecular dynamics method. *Journal of Applied Physics* **1981,** *52* (12), 7182-7190.
41. Amadei, A.; Linssen, A. B. M.; Berendsen, H. J. C., Essential dynamics of proteins. *Proteins: Structure, Function, and Bioinformatics* **1993,** *17* (4), 412-425.
42. Monti, M.; Stener, M.; Aschi, M., A computational approach for modeling electronic circular dichroism of solvated chromophores. *Journal of Computational Chemistry* **2022,** *43* (30), 2023-2036.
43. Humphrey, W.; Dalke, A.; Schulten, K., VMD: Visual molecular dynamics. *Journal of Molecular Graphics* **1996,** *14* (1), 33-38.
44. Aikens, C., Effects of Core Distances, Solvent, Ligand, and Level of Theory on the TDDFT Optical Absorption Spectrum of the Thiolate-Protected Au25 Nanoparticle The *Journal of Physical Chemistry A* **2009,** *113* (40), 10811-10817
45. Zhu, M., Aikens, C., Hollander, F.J., Schatz. G.C., Rongchao, J., Correlating the Crystal Structure of A Thiol-Protected Au25 Cluster and Optical Properties The *Journal of the American Chemical Society* **2008,** *130* (18), 5883-5885
